# Current Situation of Acute Rheumatic Fever and Rheumatic Heart Disease in Latin America and the Caribbean: A Systematic Review

**DOI:** 10.5334/gh.1152

**Published:** 2022-09-02

**Authors:** Maria Alejandra Jaimes-Reyes, Manuel Urina-Jassir, Manuel Urina-Triana, Miguel Urina-Triana

**Affiliations:** 1 Fundación del Caribe para la Investigación Biomédica, Carrera 50 # 80–216 Office 201, Barranquilla, Atlántico, Colombia; 2 Facultad de Ciencias de la Salud, Universidad Simón Bolívar, Carrera 59 # 59–65, Barranquilla, Atlántico, Colombia

**Keywords:** rheumatic heart disease, rheumatic fever, epidemiology, Latin America, Caribbean region

## Abstract

**Background:**

Rheumatic heart disease (RHD) disproportionately affects low-income and middle-income countries. Latin America and the Caribbean (LAC) have been less represented in scientific literature. We aimed to describe the epidemiology, burden and implemented screening and prevention strategies of RHD in LAC.

**Methods:**

We systematically searched PubMed, Embase, LILACS, and SciELO from 1990 to April 2021. Observational and experimental studies that described data on the epidemiology, burden, or prevention/screening strategies of RHD, regardless of age or language, were included. The risk of bias was assessed by previously published tools depending on their study design. Pre-specified data were independently extracted and presented by each topic (epidemiology, burden, prevention/screening). PROSPERO registration number: CRD42021250043.

**Results:**

Forty-eight studies out of 1692 non-duplicate records met the eligibility criteria. They were mainly from Brazil, observational in design, and hospital-based. Data on the epidemiology of acute rheumatic fever (ARF) was not recent (most before 2000) with studies describing decreasing incidence through the years. The prevalence of RHD was described in six studies, ranging from 0.24 to 48 per 1,000 among studies evaluating schoolchildren. Nine studies described data based on admissions, ranging from 0.04% to 7.1% in single-center studies. Twenty-four studies assessed the burden of RHD with most of them reporting mortality rates/proportions and complications such as the need for intervention, atrial fibrillation, or embolism. Six preventive strategies were identified that included educational, register-based, and/or secondary prophylaxis strategies. Three well-established echocardiographic screening studies in Brazil and Peru were identified.

**Conclusions:**

Most ARF/RHD research in LAC comes from a single country, Brazil where preventive/screening efforts have been conducted. There was a paucity of data from several countries in the region, reflecting the need for epidemiological studies from more countries in LAC which will provide a better picture of the current situation of ARF/RHD and guide the implementation of preventive strategies.

## 1. Introduction

Rheumatic heart disease (RHD) is the resulting chronic valvular damage after a single severe or multiple episodes of acute rheumatic fever (ARF) [1,2]. The prevalence of RHD has been increasing since 1990 according to data of the *Global Burden of Cardiovascular Diseases 2019*, with estimates of up to 40.5 million people affected in 2019 [3]. On a positive note, however, the global age-standardized mortality from RHD decreased from 9.2 deaths per 100,000 population in 1990 to 4.8 in 2015 [4]. Despite this, there still exists heterogeneity in the prevalence and mortality rates among countries or regions. For instance, in 2015, 73% of the global cases were concentrated in five countries (India, China, Pakistan, Indonesia, Democratic Republic of Congo), whereas the highest age-standardized death rates occurred in Oceania, South Asia, and central sub-Saharan Africa, indicating an unequal burden of this disease throughout the world [4].

In the Americas, a region including North America (the United States and Canada) and Latin America and the Caribbean (LAC), the prevalence and mortality estimates due to RHD in 2017 were lower than in the global population, according to a secondary analysis of the *Global Burden of Disease (GBD) 2017* study [5]. However, the age-standardized prevalence in 2017 of the LAC subregion (532.8 [513.2–552.8] per 100,000 population) was higher than that of the Americas region as a whole (346.4 [334.1–359.2]) or the global prevalence (500.6 [482.9–519.7]). Nevertheless, there was a reduction in mortality due to RHD in both, the Americas (–48.3%) and the LAC subregion (–59.0%) from 1990 to 2017 with lower mortality age-standardized estimates in 2017 (1.8 [1.7–1.9] and 1.2 [1.2–1.3] per 100,000, respectively) than the global population (3.7 [3.4–3.9]) [5]. In line with the worldwide trends, the burden of premature mortality in the Americas was also described to affect predominantly poorer countries [5]. It is important to note that the GBD studies provide essential estimates for the global patterns, but they have inherent limitations as there exists a lack of quality data from many countries, especially low-and-middle-income, including those in LAC [4,5].

Despite the global decreased tendency in the number of deaths, RHD continues to burden many low- and middle-income countries due to its long-term cardiovascular complications including heart failure (HF), pulmonary hypertension (PH), atrial fibrillation (AFib), infective endocarditis (IE), and stroke [6,7]. Multiple health organizations and world-renowned groups of experts have published recommendations and strategies that aim to reduce the burden of RHD worldwide [8,9,10]. The World Heart Federation (WHF) established the goal of reducing 25% of premature deaths from ARF and RHD by 2025 [8]. Consistent with this, the World Health Organization (WHO) Assembly approved a resolution to raise the profile of RHD on the global agenda [9]. Ordunez et al. have raised awareness about this disease in the Americas and LAC, which are regions that are less represented in the published literature on RHD [5]. Considering this, our systematic review aimed to describe the epidemiology of ARF and RHD, the burden of RHD, and the implemented screening and prevention strategies in LAC.

## 2. Methods

This review was reported in accordance with the Preferred Reporting Items for Systematic Reviews and Meta-Analysis statement (PRISMA) guidelines [11]. The protocol was registered a priori on PROSPERO (CRD42021250043) [12]. The objectives of the review were 1) to describe the epidemiology (e.g., prevalence or incidence) of ARF/RHD, 2) to describe the burden (e.g., complications, need for intervention, or mortality) of RHD, 3) to describe the screening and prevention strategies for RHD, in LAC. In addition, we also aimed to identify possible gaps in the RHD literature in LAC.

### 2.1. Information sources and search strategy

We conducted a systematic literature search in Embase, PubMed, the Latin American and Caribbean System on Health Sciences Information (LILACS, for its acronym in Spanish), and SciELO databases for studies of Acute Rheumatic Fever and/or Rheumatic Heart Disease in Latin America and the Caribbean from 1990 to April 22^nd^, 2021. We restricted the search to 1990 and onward to obtain information about ARF/RHD in LAC published in the last 30 years. No language restriction was applied. The search strategy is available in Supplementary Table S1-4.

### 2.2. Inclusion and exclusion criteria

Our inclusion criteria included the following: 1) population: subjects of any age from LAC countries, 2) condition: diagnosis of ARF and/or RHD, 3) outcomes/data reported: epidemiologic data (e.g., prevalence, incidence, or admissions), burden data (e.g., morbidity, mortality, or costs), and/or the description of screening/preventive strategies, 4) study designs: primary study designs such as experimental (randomized controlled and non-randomized trials) and observational studies (cross-sectional, cohort, and case-control studies), 5) publication year range: 1990 to April 2021, and 6) language: no restriction. Case reports and series, review articles, systematic reviews/meta-analyses, and guidelines were excluded. International studies that included data not divided by region or country, articles with duplicate information from other reports, and studies based on autopsies or necropsies were also excluded. When encountered with manuscripts on heart or valve diseases, they were included only if outcomes (e.g., prevalence, mortality, etc.) were divided by etiology (i.e., RHD). As being the first systematic review, to our knowledge, addressing these topics of RHD in LAC, we decided to have relatively broad inclusion criteria regarding study design and setting to better capture the status of RHD in this region.

### 2.3. Selection process

Two authors (MAJR and MUJ) independently assessed all records by title and abstract. Then, records were reviewed in full text and selected independently by the same two reviewers according to the eligibility criteria using Rayyan® [13]. Discrepancies between the two reviewers were determined by a consensus-based discussion or by a third reviewer (MUT) if required.

### 2.4. Data collection process

The following items were extracted from each study (if available): 1) general information (author, year of publication, study design and period, country, objectives); 2) population information (study sample, number of participants, gender, and age); 3) diagnostic criteria used for ARF and/or RHD, 4) epidemiologic characteristics (incidence, prevalence, and hospitalization/admission frequency data); 5) burden of RHD (mortality, costs of disease, need for intervention, need for anticoagulation, and complications of RHD [AFib, IE, embolic events (EE) or stroke, HF, PH, and anticoagulated-related complications]); 6) prevention and screening strategies for RHD (type of strategy, description, duration, and results of strategy). All data were independently extracted by two reviewers (MAJR and MUJ) using a spreadsheet with a prespecified extraction form with all the information stated above. Disagreements between the two reviewers were discussed; a final decision was made by mutual consensus or by a third reviewer (MUT).

### 2.5. Study risk of bias assessment

Two reviewers (MAJR and MUJ) independently evaluated the risk of bias for each study. We evaluated the risk of bias or quality of each of the included studies depending on each of their study design. The assessment tools used were the Newcastle-Ottawa Scale (NOS) [14] for cohort studies, the AXIS Critical Appraisal Tool for Cross-Sectional Studies [15], and the Cochrane Revised Tool for Risk of Bias in randomized controlled trials (RoB 2.0) [16] (and its extension for Cluster Randomized Trials [17]). Disagreements were resolved by discussion between authors (MAJR and MUJ) or by a third reviewer (MUT). We did not exclude any studies based on quality assessment.

### 2.6. Synthesis methods

Due to the high heterogeneity among the studies, we conducted a narrative synthesis divided by each topic: epidemiology of ARF/RHD, the burden of RHD, and prevention and screening strategies of ARF/RHD.

## 3. Results

### 3.1. Selection of studies

**Figure 1:**
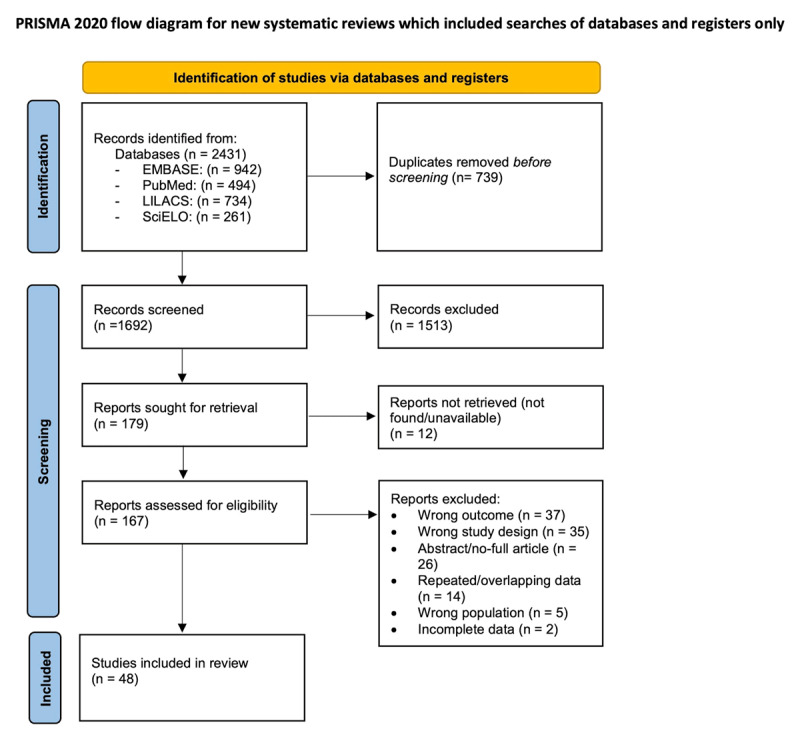
PRISMA [11] flow diagram for study selection.

Our search yielded a total of 2431 records of which 1692 were screened by title/abstract after duplicates were removed. One hundred seventy-nine records were sought for retrieval and ultimately 48 studies fulfilled the eligibility criteria [18,19,20,21,22,23,24,25,26,27,28,29,30,31,32,33,34,35,36,37,38,39,40,41,42,43,44,45,46,47,48,49,50,51,52,53,54,55,56,57,58,59,60,61,62,63,64,65]. A PRISMA [11] flow diagram of our search strategy and reasons for the exclusion of full-text articles can be seen in Figure 1.

### 3.2. Study characteristics

Studies were from Barbados (n = 1), Brazil (n = 31), Chile (n = 3), Cuba (n = 1), Dominican Republic (n = 1), Guatemala (n = 1), Jamaica (n = 1), Martinique/Guadeloupe (n = 1), Mexico (n = 2), Nicaragua (n = 1), Peru (n = 2), Uruguay (n = 1), and Venezuela (n = 1). Additionally, one study included Bolivia, El Salvador, and Jamaica as the ‘Americas’ [64]. They were published between 1990 and 2021 with most studies published during or after 2010 (n = 26).

Data on epidemiology, burden, and prevention/screening strategies were extracted from 23, 24, and 11 studies, respectively. Regarding the setting, studies were hospital-based (n = 28), school-based (n = 5), community-based (n = 3), or population-based (n = 8). In addition, four studies evaluated schoolchildren in addition to another setting (hospital or community). Supplementary Table S5 describes the general characteristics of each reference.

### 3.3. Risk of bias among included studies

#### 3.3.1. Cross-sectional studies

Among the 30 cross-sectional studies, AXIS scores ranged from 8 to 20. Most studies lack sample size justification (Question 3) and non-responders’ characterization (Question 7). Other common flaws were lack of description of statistical significance (Question 10) or limitations acknowledgment in the discussion section (Question 18) (Supplementary Table S6).

#### 3.3.2. Cohort studies

The NOS scores of the 16 cohort studies ranged from five to nine stars. Those studies with a higher risk of bias were due to not describing adequately the ‘Comparability of cohorts’ as indicated in the NOS. Individual scores are presented in Supplementary Table S7.

#### 3.3.3. Randomized controlled trials

Two randomized controlled trials (RCTs) were included. Both RCTs were found to have an overall risk of bias of ‘Some Concerns’. None had ‘High Risk of Bias’ in any section; a detailed assessment for each section is presented in Supplementary Table S8.

### 3.4. Epidemiology of ARF and RHD in Latin America and the Caribbean

Twenty-three studies assessed epidemiological data of patients with either ARF, RHD, or both. Fifteen of them presented incidence or prevalence data whereas nine studies evaluated admissions-based data (one study assessed both). Tables 1 and 2 summarize the epidemiological data per study.

**Table 1:** Prevalence and incidence of acute rheumatic fever and rheumatic heart disease in Latin America and the Caribbean.


REFERENCE	COUNTRY	SETTING	TARGET POPULATION	DIAGNOSTIC CRITERIA	PERIOD	PREVALENCE	INCIDENCE

***Acute Rheumatic Fever***							

Noah, 1994 [47]	Barbados	Population	Children Total	Jones	1971–1990	NR	**Total population:** 1971–1972: 13/100,000 1973: 12/100,000 1974: 5/100,000 1975: 8/100,000 1976–1977: 7/100,000 1978: 9/100,000 1979: 8/100,000 1980: 5/100,000 1981: 3/100,000 1982–1984: 5/100,000 1985: 3/100,000 1986–1990: 2/100,000 **Childhood population (<19 years)**‘Since 1986’: 8/100,000

Alves Meira et al., 1995 [18]	Brazil	School	10–20 years	Jones	1992	3.6/1,000	NR

Berrios et al., 1993 [21]	Chile	Community	N/A	Jones	1982–1986	NR	1982–1985: 22.5 per year 1986(6–14-years): 21.7/100,000

Luque et al., 2006 [38]	Chile	Population	N/A	N/A	1978–1998	NR	1978: 2.2/100,000 1979: 3.2/100,000 1980: 1.4/100,000 1981: 1.6/100,000 1982: 2.4/100,000 1983: 3/100,000 1984: 2.5/100,000 1985: 2/100,000 1986: 1.9/100,000 1987: 1.3/100,000 1988–89: 1/100,000 1990: 0.6/100,000 1991–92: 0.5/100,000 1993: 0.3/100,000 1994–95: 0.2/100,000 1996–97: 0.1/100,000 1998: 0/100,000

Nordet et al., 2008 [48]	Cuba	School	5–14 years	Inactive RF: ‘History of ARF without established RHD’	1985, 1996	1985: 1.75/1,000 1996: 5.78/1,000	*See below in ARF/RHD section for incidence*

Bach et al., 1996 [19]	Martinique Guadeloupe	School Hospital	<20 years	Jones	1982–1983	NR	**Martinique:** 19.6/100,000 **Guadeloupe:** 17.4/100,000

Soto Lopez et al., 2001 [58]	Mexico	Population	5–20 years	Jones	1994–1999	NR	‘Annual incidence tendency decreased from 1.3% to 0.3%’

***Rheumatic Heart Disease***							

Meira et al., 2005 [39]	Brazil	Hospital	Children Adolescent	Echo^2^	1983–1998	NR	186 (72.1%) – ***Severe:*** 41 (15.9%) *out of 258 with ARF*

Miranda et al., 2014 [43]	Brazil	School	Children Adolescent	Auscultation Echo (WHO)	2010–2011	**Clinical:** AR: 3.7/1,000 MR: 3.7/1,000 **Echo:** AR: 7.5/1,000 MR: 18.7/1,000	NR

Nascimento et al., 2018 [46]	Brazil	School Primary care centers	Children Adolescent	Echo (WHF)	2014–2016	**Borderline RHD:** 4% (478/12,048) **Definite RHD:** 0.5% (63/12,048)	NR

Nascimento et al., 2021 [45]	Brazil	Community	Pregnant	Echo (ASE-REWARD study)	2018–2019	**Hand-held echo screening:** 3.2% (36/1,112) **Standard echo:** 1.2% (12/1,112)	NR

Nordet et al., 2008 [48]	Cuba	School	5–14 years	Echo^3^	1985, 1996	1985: 2.27/1,000 1996: 0.24/1,000	***See below in ARF/RHD section for incidence***

Paar et al., 2010 [51]	Nicaragua	Community	Children Adult	Echo (WHO)	2006–2009	**Pediatric:** 48/1,000 **Adult:** 22/1,000	NR

Spitzer et al., 2015 [60]	Peru	School	Children Adolescent	Echo (WHO & WHF)	2014	**WHO:** 19.7/1,000 children **WHF:** 3.9/1,000 children	NR

***Acute Rheumatic Fever/Rheumatic Heart Disease*** ^1^							

Souza et al., 1990 [59]	Brazil	School Community	Children Adolescent	Jones	N/A	20.3% (198/972)	NR

WHO Cardiovascular Diseases Unit, 1992^4^ [64]	Bolivia El Salvador Jamaica (Americas)	School	Children	N/A	1986–1990	**Americas:**1.5 (0.1–7.9)/1,000 **Bolivia:** 7.9/1,000	NR

Nordet et al., 2008 [48]	Cuba	Population (Incidence)School (Prevalence)	5–25 years	Inactive RF: ‘History of ARF without heart valve damage’RHD: Echo^3^	1986, 1996, 2002	**5–14 years:** 1985: 8.01/1,000 1996: 1.99/1,000	**5–25 years:** 1986: 18.6/100,000 1996: 2.5/100,000 2002: 2.4/100,000 **5–14 years** 1986: 28.4/100,000 1996: 2.7/100,000 2002: 2.8/100,000


^1^ Studies that reported the epidemiologic data combining both terms ARF and RHD or referred to them as ‘ARF/RHD’. ^2^ Reported as ‘the Doppler echocardiography criteria adopted by the echo lab of Universidad Federal Minas Gerais’ [39]. ^3^ Reported as ‘typical RHD valve damage supported by echocardiogram’ [48]. ^4^ The manuscript includes data from 16 countries divided into 5 regions; only data of the Americas region was extracted. **Abbreviations:** ASE: American Society of Echocardiography; AR: Aortic regurgitation; ARF: Acute rheumatic fever; ICD: International Classification of Diseases; MR: Mitral regurgitation; N/A: Not available; NR: Not reported; RF: Rheumatic fever; RHD: Rheumatic heart disease; WHF: World Heart Federation; WHO: World Health Organization.

**Table 2:** Admissions-based data of acute rheumatic fever and rheumatic heart disease in Latin America and the Caribbean.


REFERENCE	COUNTRY	TARGET POPULATION	PERIOD	DIAGNOSTIC CRITERIA	N/N (%)	DESCRIPTION

***Acute Rheumatic Fever***						

Silva et al., 2010 [57]	Brazil	Children Adolescent	1986, 1991, 1996, 2001, 2006	Jones	1986: 59/4206 (1.4%) 1991: 17/5206 (0.3%) 1996: 8/5196 (0.15%) 2001: 12/6777(0.18%) 2006: 3/8203 (0.04%)	# of ARF admissions/# of admissions in each period in a single pediatric center

de Araújo Fonseca et al., 2020 [26]	Brazil	N/A	2008–2017	ICD-10	42,720/11,345,821 (0.4%)	# of ARF admissions/# of CVD admissions in Brazil 2008–2017

Defilló Ricart et al., 1991 [27]	Dominican Republic	Children	1969–1989	Jones	121/19,483 (0.62%)	# of ARF cases/# of admissions in Cardiology Department of Pediatric Hospital

Stokes Baltazar, 2007 [61]	Guatemala	Children AdolescentAdult	2000–2005	Jones	246/3422 (7.1%)	# of ARF cases/# of admissions from a single center

Millard-Bullock, 2012 [42]	Jamaica	Children	1975–19851989–1995	Jones	1975–1985: 54% (total pop.: 1079) 1989–1995: 55% (total pop.: 512)	% of patients with ARF among children admitted to hospitals in Jamaica (1975–1985: 4 hospitals, 1989–1995: 3 hospitals)

Soto Lopez et al., 2001 [58]	Mexico	Children Adolescent	1994–1999	Jones	**Incidence:** 6.6 per 1,000 (Total pop.: 3392)	Incidence of new ARF cases out of the total admissions among 5–20-year-olds in a single Cardiology center

Giachetto et al., 1994 [31]	Uruguay	Children Adolescent	1990–1993	Jones	1990: 14/1731 (0.82%) 1991: 8/2032 (0.39%) 1992: 18/2063 (0.87%) 1993: 18/2256 (0.79%)**Total:** 58/8,082 (0.71%)	# of ARF admissions/# of children aged 2–14 admissions in a single pediatric center

***Rheumatic Heart Disease***						

Haddad and Bittar, 2005 [32]	Brazil	N/A	1988–2003	ICD-9 (1988–94)ICD-10	**Men:** 3.1% **Women:** 9.8%	Mean relative percentage per month of RHD diagnosis out of the total admissions in a single CVD center

de Araújo Fonseca et al., 2020 [26]	Brazil	N/A	2008–2017	ICD-10	78,966/11,345,821 (0.7%)	# of RHD admissions/# of CVD admissions in Brazil 2008–2017

***Acute Rheumatic Fever/Rheumatic Heart Disease*** ^1^						

Salinas Mondragón et al., 1995 [54]	Peru	Children Adolescent	1989–1993	Jones	1989: 9/174 (5.1%) 1990: 10/215 (4.6%) 1991: 16/177 (9.0%) 1992: 15/263 (5.7%) 1993: 16/245 (6.5%) **Total:** 66/1074 (6.1%)	# of hospital discharges with ARF/RHD/# discharges in a single pediatric center


^1^ Studies that reported the epidemiologic data combining both terms ARF and RHD or referred to them as ‘ARF/RHD’. **Abbreviations:** ARF: Acute rheumatic fever; CVD: cardiovascular diseases; ICD: International Classification of Diseases; N/A: Not available; RHD: Rheumatic heart disease.

#### 3.4.1. Prevalence of ARF and RHD in Latin America and the Caribbean

Data on the prevalence of each study are described in Table 1. Two studies from Brazil [18] and Cuba [48] reported the prevalence of ARF with one using Jones Criteria for case definition [18]. The prevalence in the Brazilian study was 3.6 per 1,000 among schoolchildren from Belo Horizonte in 1992 [18]. Whereas Nordet et al. described a prevalence of 1.75 and 5.78 per 1,000 in 1985 and 1996, respectively, in a Cuban study [48]. On the other hand, six studies evaluated data on RHD [43,45,46,48,51,60], whereas three from Brazil, Cuba, and the Americas region described epidemiological data combining both terms (ARF/RHD) [48,59,64]. The latter corresponds to data collected by the World Health Organization program from 1986 to 1990 in Bolivia, El Salvador, and Jamaica, where a prevalence of 1.5 per 1,000 (ranging from 0.1 to 7.9 among the countries) was identified [64].

The prevalence of RHD was described in three Brazilian studies [43,45,46] as well as studies from Cuba [48], Nicaragua [51], and Peru [60]. All studies based their diagnosis on echocardiographic criteria; two of the Brazilian reports were based on the Rheumatic Valve Disease Screening Program (PROVAR) and its extension (PROVAR+) [45,46]. The population assessed was mostly focused on the non-adult population, whereas one study evaluated pregnant women [45] and another study included adults in addition to the pediatric population [51]. The prevalence among studies assessing children or adolescent population ranged from 0.24 per 1,000 (Cuba, 1996) [48] to 48 per 1,000 (Nicaragua, 20062009) [51]. Furthermore, the prevalence of RHD among pregnant women in the primary care setting in Minas Gerais, Brazil was 1.2% [45].

#### 3.4.2. Incidence of ARF and RHD in Latin America and the Caribbean

Table 1 presents the incidence of ARF and RHD per study. Most studies reported the incidence of ARF; these were from Barbados, Chile, Martinique, Guadeloupe, and Mexico [19,21,38,47,58]. All except for one, where the definition was not described [38], used Jones criteria for case definition. When data from more than one year was available, the studies from Barbados, Chile, and Mexico described a lower incidence throughout their study years [38,47,58]. For instance, the incidence of ARF in Chile decreased from 2.2 per 100,000 in 1978 to 0 in 1998 [38] and in Barbados from 13 per 100,000 in 1971 to 2 per 100,000 in 1990 [47]. No data from the 2000s, 2010s, and 2020s were identified about ARF incidence. One study prospectively followed Brazilian children and adolescents with ARF to evaluate the progression to RHD; these authors found that 72.1% (186/258) developed chronic disease [39]. Moreover, one study assessed the incidence of combined ARF/RHD in two different age groups in Cuba, identifying a decreasing incidence from 1986 to 2002 [48].

#### 3.4.3. Admissions-based data on ARF and RHD in Latin America and the Caribbean

Data on admissions or discharges of ARF, RHD, or both were assessed in nine studies from Brazil, Dominican Republic, Guatemala, Jamaica, Mexico, Peru, and Uruguay [26,27,31,32,42,54,57,58,61]. Most of them were based on single-center experiences except for two studies [26,42]. A Brazilian study evaluated all the cardiovascular admissions in the country over 10 years, identifying that 0.4% and 0.7% of them were due to ARF and RHD, respectively [26]. Furthermore, Millard-Bullock assessed the data from three and four Jamaican hospitals in two study periods, 1975–1985 and 1989–1995, respectively, with a high frequency of admitted patients with ARF (54% and 55%, respectively) [42]. Among single-center studies on either ARF or ARF/RHD, the percentage of patients with this diagnosis ranged from 0.04% (Brazil, 2006) [57] to 7.1% (Guatemala, 2000–2005) [61]. A summary of the results of these investigations is presented in Table 2.

### 3.5. Burden of RHD in Latin America and the Caribbean

Twenty-four studies described data on the burden of RHD. Fifteen reports were based on solely surgical or percutaneously intervened subjects whereas the other studies included subjects with RHD regardless of their treatment (general studies).

#### 3.5.1. Mortality of RHD

Seven studies reported RHD mortality regardless of their treatment; five were from Brazil [30,33,37,49,63], while the other two were from Peru [54] and Venezuela [34]. Studies reported data with different measures (e.g., proportions, rates) with four of them reporting mortality rates. Two of them assessed mortality rates among women of reproductive age in Brazil; Lolio et al. reported a mortality rate of 2.6 per 100,000 women in 1986 while Haddad and Silva described 1.58 per 100,000 women from 1991 to 1995 [33,37]. More recently, Figueiredo et al. utilized data from the Brazilian health system and described RHD mortality rates of 5.77 and 8.22 in 1998 and 2016, respectively [30]. A Venezuelan cross-sectional study reported adjusted mortality rates from data from the Health Ministry, with rates per 100,000 declining from 7.06 in 1955 to 1.05 in 1994 [34]. From the studies reporting the proportion of demised patients, this ranged from 0.8% to 6% [49,54,63]. Further data on mortality among general studies are presented in Table 3 and Supplementary Table S9.

**Table 3:** Burden of rheumatic heart disease in Latin America and the Caribbean*.


*COMPLICATION*	*REPORTED DATA AND REFERENCES*	

	General studies^1^	Intervention-only studies^1^

**Mortality**	**Rates** BR: 2.6/100,000 women (1986) [37] 1.58/100,000 women (1991–1995) [33] 5.77 (1998), 8.22 (2016) [30] VE: 7.06 (1955), 3.04 (1966), 0.78 (1975), 1.66 (1985), 1.05 (1994)/100,000 [34] **Proportions** BR: 0.8% (2007–2011) [49] 6.2% (2010–2019) [63] PE: 6% (1989–1993) [54]	**Operative:** BR: 0% (1994–2005) [56] 2.7% (1996–2005) [62] 13% (2008–2009) [22] CL: 9.4% (1990–2004) [55] **In-hospital or <30 days:** BR: 5.4% (1991–1994) [35] 0% (1994–2005) [56] 9% (2002–2005) [53] 19.2% (2007–2011) [29] 10% (2010–2011) [52] 7.8% (2013–2014) [24] 3.51% (2010–2015) [40] **Follow-up:** BR: 2-month: 0% (2011–2017) [25] 3-month: 0% (2010–2012) [28] 1-year: 0% (2013–2014) [24] 38.5–41.1-month: 7.3% (1991–1994) [35] 63 ± 39-month: 2.9% (1994–2005) [56] CL: 6.67–7.89-years: 17.7% (1990–2004) [55] **Overall** BR: 0.6% (1987–2010) [41] 8.2% (1996–2005) [62] MX: 20% [65]

**Need for intervention** ^2^	**At baseline** BR: 27% (2007–2011) [49] 25% (2010–2019) [63] **During follow-up** BR: 34.4% (2007–2011) [49] 21.5% (2010–2019) [63] **Overall** CU: 4.5% (1986–1990), 0.5%(1991–1996) [48] PE: 12.1% (1989–1993) [54]	**At baseline** BR: 30% (2002–2005) [53] 38% (2007–2011) [29] 63% (2010–2011) [52] **Reintervention** BR: 11.5% (1994–2005) [56] 12.7% (1996–2005) [62] 23.07% (2007–2011) [29] 8.3% (Surgery), 10% (PBMV) (1987–2010) [41] 10% (2010–2011) [52] 27.9% (First), 14.8% (Second) (2010–2015) [40] 5.6% (2011–2017) [25] CL: 4.7% (1990–2004) [55]

**Heart failure**	CU: 11.2% (1986–1990), 1.5% (1991–1996) [48]	BR: 22.3% (2009) [23] 7.4% (Postop.) (2011–2017) [25] CL: 5.1% (1990–2004) [55]

**Atrial fibrillation**	BR: 14% (2007–2011) [49] 30% (2010–2019) [63]	BR: 12.5% (1987–2010) [41] 28% (2007–2011) [29] 53.1% (2009) [23] 0% (2011–2017) [25] CL: 65.6% (Preop.), 63.3% (Postop.) (1990–2004) [55]

**Infective endocarditis**	CU: 0% (1986–1996) [48] PE: 23% (1989–1993) [54]	BR: 2.8% (1996–2005) [62] 1.9% (2008–2009) [22] 16% (2010–2011) [52] CL: 1.4% (1990–2004) [55] MX: 7.1% [65]

**Stroke**	BR: 18% (Baseline), 5.2% (Follow-up) (2010–2019) [63] 12.7% [36] PE: 1.5% (1989–1993) [54]	BR: 4.2% (1996–2005) [62] 7.5% (2008–2009) [22] 2.7% (2010–2012) [28] 10.5% (Baseline), 1% (Postop.) (2013–2014) [24] CL: 2.8% (1990–2004) [55]

**Embolic events**	BR: 4.4% [36]	BR: 16.4% (Baseline), 12.7% (Postop.) (1991–1994) [35] MX: 7.1% [65]

**Pulmonary hypertension**	PE: 16.7% (1989–1993) [54]	BR:57.5% (2009) [23] 77.6% (Preop.), 18.4% (Postop.) (2011–2017) [25]


* Supplementary Table S9 includes the information on burden of RHD per each included study. ^1^ Several studies (‘Intervention-only studies’) that assessed solely surgical or percutaneously intervened RHD patients while others (‘General studies’) assessed patients receiving any kind of treatment. ^2^ ‘Need for intervention’ includes any surgical (initial or reoperation) or percutaneous intervention (initial or reintervention). **Abbreviations:** BR: Brazil, CL: Chile, CU: Cuba, MX: Mexico, PBMV: Percutaneous Balloon Mitral Valvuloplasty; PE: Peru, Postop.: postoperative, Preop.: preoperative, VE: Venezuela.

Among the studies assessing surgically or percutaneously intervened, thirteen were from Brazil, one from Chile [55], and one from Mexico [65]. All, except one [23], reported data on mortality, which timeframes varied among studies. Among those reporting operative mortality, proportions ranged from 0 to 13% [22,55,56,62], whereas in those reporting in-hospital or <30 days mortality, it ranged from 0% to 19.2% [24,29,35,40,52,53,56]. Some authors reported distinct follow-up periods for longer-term mortality (from 2 months up to 8 years of follow-up) [24,25,28,35,55,56] whereas others reported overall mortality during their total study period ranging from 0.6% to 20% [41,62,65]. Moreover, Ribeiro et al. reported the mean annual incidence of in-hospital mortality (0.25 per 100,000) and of open-heart surgery for RHD (2.86 per 100,000) in Salvador, Brazil [53] (Table 3 and Supplementary Table S9).

#### 3.5.2. Morbidity of RHD

Among general studies, burden was represented as the need for an intervention [48,49,54,63], need for anticoagulation [63], HF [48], AFib [49,63], IE [48,54], stroke or EE [36,54,63], and PH [54]. Regarding the need for surgery or percutaneous intervention, this varied among studies from 0.5% [48] to 34.4% [49]. Two studies reported that patients with RHD had concomitant AFib in 14% and 30%, respectively [49,63]. Lavitola et al. reported an incidence of EE of 3.7% patient/year in an RCT of warfarin versus aspirin among RHD patients [36]. Recently, Vasconcelos et al. reported an incidence of 1.47 strokes per 100 patient-years in a prospective cohort study among Brazilian adults with RHD [63]. The proportion of other complications (with more than one study reporting the variable) ranged from 0%–23% for IE [48,54] and 1.5%–18% for stroke/EE [36,54,63]. Information on the burden data per study is shown in Table 3 and Supplementary Table S9.

Burden was also assessed from surgical or percutaneously intervened studies. The need for reoperation or reintervention was commonly described in the studies [25,29,40,41,52,55,56,62]. Other complications of RHD reported among interventional studies were HF (5.1%–22.3%) [23,25,55], AFib (0%–65.6%) [23,25,29,41,55], IE (1.4%–16%) [22,52,55,62,65], stroke (2.7%–10.5%) [22,24,28,55,62], EE (7.1%–16.4%) [35,65], and PH [23,25]. Table 3 and Supplementary Table S9 present data on burden per study.

#### 3.5.3. Economic impact of RHD

Three studies assessed the economic consequences of RHD in LAC countries [30,42,48]. In Pinar del Rio, Cuba, the estimated mean cost per year due to ARF/RHD care was $97,457.00 USD from 1986 to 1996 [48]. Figueiredo et al. reported that the total costs due to RHD increased from $7,006,288.21 USD (1998) to $25,526,924.01 USD (2016) in Brazil [30]. Furthermore, Millard-Bullock commented on a total hospitalization cost for ARF/RHD of $17 million Jamaican Dollars (JMD) per year among the three hospitals assessed in 1989–1995 in Jamaica [42].

### 3.6. RHD prevention and screening strategies in Latin America and the Caribbean

#### 3.6.1. RHD prevention programs in Latin America and the Caribbean

Different prevention programs have been launched in Brazil [44], Chile [21], Cuba [48], Jamaica [42], Martinique, Guadeloupe [19], Bolivia, El Salvador, and Jamaica [64]. The study periods of these strategies ranged from 1975 to 2001. Four of these strategies included case finding or registries of patients with ARF/RHD [19,42,48,64]. Chemoprophylaxis for secondary prevention was a part of five of the prevention programs [21,42,44,48,64]. Another common aspect of the projects was the education of the general population, teachers, and/or healthcare workers [19,42,48,64]. Three of these strategies described a decrease in unfavorable outcomes including cases/recurrences [19,44,48], severity [48], hospital admissions [44], surgeries [19,44], deaths [44], and costs [19,48]. The specific description and the results obtained during each program are presented in Table 4.

**Table 4:** Preventive and screening strategies for rheumatic heart disease in Latin America and the Caribbean.


REFERENCE	COUNTRY	STUDY PERIOD	DESCRIPTION	RESULTS

***Prevention programs***				

Mota et al., 2015 [44]	Brazil	1977–2000	**Prevention Program for ARF-UFMG (since 1988)** HCW education. Outpatient clinic for ARF with routine follow-up. Echocardiographic screening for definite or suspected cases of ARF or RHD. Distribution of free medication. Chemoprophylaxis and ‘prophylaxis card’ to control compliance. Active searching of missing patients.	Comparing two periods (July 1977–July 1988, n = 248 and August 1988–February 2000, n = 454), the authors identified a decrease in: Recurrences (22.4 vs 7.4, p = 0.0000) Hospital admissions (45.4 vs 28.4, p = 0.0000) Surgeries (13.3 vs 1.5, p = 0.0000) Deaths (5.4 vs 0.4, p = 0.0000)

Berrios et al., 1993 [21]	Chile	1982–1988	**ARF Control and Prevention Program of Southeast Health District (Catholic University Medical School, Santiago, Chile)** Chemoprophylaxis: benzathine penicillin G every 28 days (nonpenicillin allergic) or oral sulfadiazine daily (penicillin allergic) Duration: no carditis: 5 years or until age 18; carditis: 10 years or until age 25; aortic involvement, mitral stenosis or multivalvular: for life. Follow-up after cessation: regular visits every 3 months, annual examination by cardiologist.	59 post prophylactic patients (1032 scheduled visits and 3346 patient- months) Recurrence rate of 0.72 (CI, 0.2 to 2.6) per 100 patients-years of prospective surveillance.

Nordet et al., 2008 [48]	Cuba	1986–2001	**Pinar del Rio Project** Primary prevention of ARF/RHD: diagnosis and treatment of GAS pharyngitis. Secondary prevention of ARF/RHD: case finding, referral, permanent register, surveillance, and chemoprophylaxis for ARF/RHD. Educational program and personnel training. Two cross-sectional studies for ARF/RHD prevalence in schoolchildren (5–15 year) were conducted in 1985 and 1996.	Decline in the occurrence and severity of RF/RHD. **(See Results section, Tables 1 and 3)** Decline of ARF/RHD incidence in schoolchildren and 5–25-year-olds. **(See Results section, Table 1)** Increase in secondary prophylaxis compliance: 1986 (50% regular, 36.5% irregular and 13.5% non-compliance) to 1996 (93.8% regular, 6.2% irregular). Decline in estimated direct costs of RF/RHD: 145519 USD per year (1986–1990) to 49376 USD per year (1991–1996).

Millard-Bullock, 2012 [42]	Jamaica	1975–1985, 1989–1995	**The ARF and RHD Control Program – Jamaica** Primary prevention of ARF (identification and treatment of GAS infections). Secondary prevention with 4-weekly injections of benzathine penicillin. Case-finding, registration, and surveillance of patients. Health education to patients, families, and the public. (Conferences, seminars, posters, etc.)	ARF cases surveys (**See Results section, Table 2)** ARF/RHD’s hospitalization cost (1989–1995): J$17 million per year for 3 hospitals.

Bach et al., 1996 [19]	Martinique Guadeloupe	1982–1992	**Martinique/Guadeloupe eradication program** Registry of all cases. Educational program (pamphlets, posters, films, etc.) on sore throat, ARF, and HCW education. Research (immunological, bacteriological, or genetic studies) on patients with ARF.	Decline of ARF cases in both islands. (78% reduction in Martinique and 74% in Guadeloupe) Decline of patients requiring open heart surgery due to rheumatic fever carditis before age 18. Cost reduction of recent childhood rheumatic fever from USD 1,426,000 to USD 100,000 (86% decrease)

WHO Cardiovascular Diseases Unit, 1992 [64]	BoliviaEl SalvadorJamaica (‘Americas’)	1986–1990	**WHO program for the prevention of ARF/RHD in 16 developing countries** The Americas region was one of the five regions assessed and included Bolivia, El Salvador, and Jamaica. The program included: Case finding via screening surveys of schoolchildren, hospital retrospective case surveys, and continuing detection/referral of any ARF/RHD confirmed or suspected case from health centers. Central register of all confirmed ARF/RHD patients. Follow-up consultation and secondary prophylaxis in local centers. Personnel (schoolteachers and HCW) training and health education.	The Americas region results: Five surveys (n: 23,328 schoolchildren) conducted for ARF/RHD prevalence **(See Results section, Table 1)** Case detection and registration; a total of 9,645 on the register (35 detected in screening, 881 from other sources and 8,729 known cases) Rate of coverage of prophylaxis: 47.2% (23.8–75.6%) per 100 patients registered for secondary prophylaxis per month. El Salvador had one of the lowest rates among all 16 countries: 23.8%. Training of personnel: 2,080 (123 doctors, 1,147 other HCW, and 819 schoolteachers) Activities for health education: pamphlets and brochures: 24,304, posters: 28, radio/TV programs: 22, and group sessions: 93.

***Screening programs***				

Beaton et al., 2016 [20] Nascimento et al., 2018 [46]	Brazil	2014–2016	**PROVAR: *Rheumatic Valve Disease Screening Program*** RHD echocardiographic screening program in Minas Gerais, Brazil. Image acquisition by nonexperts on portable and/or handheld devices. Telemedicine interpretation by experts in Brazil and USA (WHF 2012 criteria). Patients with confirmed abnormalities referred for clinical and echocardiographic follow-up. RHD educational curriculum delivered in schools and in primary care **(See Oliveira et al., 2020 below)**. Echocardiographic education of non-experts (n: 6): self-directed educational experience followed by field-testing of school-based handheld echocardiography screening [20].	Screening in 52 public schools, 2 private schools and 3 primary care centers. (**See Results Section, Table 1)** Educational curriculum delivered to 29,695 children. Non-experts’ interpretation of echocardiography: sensitivity 83% and specificity 85% for detecting RHD (borderline or definite) [20].

Nascimento et al., 2021 [45]	Brazil	2018–2019	**PROVAR+: *Programa de RastreamentO da VAlvopatia Reumatica e outras Doenças Cardiovasculares*** A continuation of PROVAR [46]. This publication involved an echocardiographic screening program among pregnant women in prenatal care: Image acquisition by nonexperts on hand-held devices. Telemedicine image interpretation by experts in Brazil and USA. HCW received educational curriculum on echocardiography. Standard echocardiogram was scheduled for those with significant abnormalities during screening.	1112 pregnant women were screened. (See Results section, Table 1) Authors concluded that integrating this type of strategies is possible in the Brazilian system.

Spitzer et al., 2015 [60]	Peru	2014	**Echocardiopraphic Screening Program on Schoolchildren at Arequipa, Peru** RHD echocardiographic screening program among schoolchidren (5–16 years) in Arequipa, Peru. Cardiac auscultation. Portable echocardiography by cardiologist. If pathologic findings, a detailed echo by local cardiologist was offered. WHO and WHF classifications were evaluated by 5 cardiologists from Bern University Hospital. Secondary prophylaxis and regular follow-up for children with borderline/definite (WHF) or probable/definite (WHO) RHD.	1023 children were screened and pathological findings on echocardiography were reported in 59 children (5.8%) and 45 underwent confirmatory echocardiogram. **(See Results section, Table 1)** 21 children (4 with concomitant RHD) had congenital heart disease. Secondary prophylaxis in six children with WHO definite/probable. RHD, and one with WHF borderline RHD.

***Educational programs/interventions***				

Oliveira et al., 2020 [50]	Brazil	2016–2017	**RHD educational strategy by PROVAR researchers** Two educational strategies were assessed by a cluster randomized trial among schoolchildren: **‘Conventional’:** classes with slide presentations provided by a research nurse. **‘Experimental’:** individual interactive modules provided in tablets.	Baseline knowledge of ARF/RHD was low. Improvement in knowledge was similar immediately after intervention. After 3 months, worsening in knowledge was observed (similar in both groups). Authors concluded that these educational strategies improve knowledge (which may be important in prevention). However, retention of knowledge was low.


**Abbreviations:** ARF: Acute rheumatic fever; GAS: Group A Streptococcus; HCW: Healthcare workers; **J$:** Jamaican Dollars; UFMG: Federal University of Minas Gerais; PROVAR: Rheumatic Valve Disease Screening Program; PROVAR+: Programa de RastreamentO da VAlvopatia Reumática e outras Doenças Cardiovasculares; RHD: Rheumatic Heart Disease; TV: Television; USD: United States Dollars; WHF: World Heart Federation; WHO: World Health Organization.

#### 3.6.2. RHD screening programs in Latin America and the Caribbean

The Rheumatic Valve Disease Screening Program (PROVAR) in Minas Gerais, Brazil, consisted of the use of handheld devices to obtain cardiac imaging among approximately 12,000 children; these images were then reviewed by experts, and those with abnormal findings were offered follow-up [46]. Additionally, as part of this program, non-experts were also educated on echocardiography [20]. Consequently, this strategy was expanded to include other populations (PROVAR+) such as pregnant women in their prenatal care [45]. An educational strategy on ARF/RHD by the PROVAR researchers was also conducted, comparing two educational methods among schoolchildren [50]. Spitzer et al. conducted echocardiographic screening among schoolchildren in Arequipa, Peru. In this project, imaging was obtained by a cardiologist and two distinct classifications were assessed by other experts abroad and those with abnormal findings were offered follow-up [60] (Table 4).

## 4. Discussion

This systematic review provides an overview of the status of the epidemiology, burden, and preventive strategies of rheumatic heart disease in Latin America and the Caribbean. Forty-eight observational and experimental studies fulfilled eligibility criteria and are presented to describe the continuous burden of this disease in the region.

### 4.1. Epidemiology

We identified relevant data of the epidemiology on ARF/RHD in LAC. As expected, the studies varied in central aspects such as study periods, case definitions, and target population. Overall, the first main finding of our review is the paucity of recent data on the incidence or prevalence of ARF; the included studies mostly assessed data collected before the 2000s. On the other hand, studies on RHD have been more constant and have been published throughout the decades. We identified echocardiographic studies conducted in Brazil [43,45,46], Nicaragua [51], and Peru [60]. These investigations were mostly targeting children and adolescents and generally utilized either the WHO or WHF echocardiographic criteria. A meta-analysis assessing echocardiographic studies in school and community settings described a global prevalence of RHD that ranged from 5.2‰ (95% CI, 3.0–8.0) to 26.1‰ (95% CI, 19.2–33.1) according to the studies’ diagnostic criteria [66]. This type of study provides two crucial aspects. First, valuable epidemiological information, and second, the opportunity to offer follow-up and secondary prophylaxis to those with RHD findings [2]. The latter is supported by the recently published GOAL trial which provides evidence that secondary chemoprophylaxis reduced the risk of progression among subjects with latent RHD in a two year follow up [67]. Despite the recent echocardiographic studies in the mentioned countries, there is still room for launching similar strategies in LAC, considering the benefits of early diagnosis and secondary prophylaxis among patients with RHD.

Furthermore, we also evaluated the hospital-based data on ARF/RHD in LAC. Most studies were based on single-center experiences and reported the frequency or percentage of ARF/RHD out of the total number of admissions. There are clear limitations to hospital-based data such as selection bias (e.g., missing of asymptomatic, or mildly symptomatic patients), variations in each hospital’s admission criteria, and patient’s access to healthcare [68]. However, data as that reported by de Araújo Fonseca et al., using a nationwide hospital admissions registry, are useful to provide an estimate on the general picture of the disease in a certain population [26]. It’s important that further studies using this type of data also assess trends of ARF/RHD throughout the years [68].

The GBD studies have generated essential estimates on RHD worldwide and in the Americas through well-established models [4,5]. Based on these data, the Americas have been identified as a region in a more favorable situation when compared to the global picture of RHD [5]. However, consistent with our findings, Ordunez et al. indicate the lack in quantity and quality of data on certain countries in the region [5]. There is a clear need for epidemiologic surveillance on these diseases in LAC, a global barrier identified by the WHF position statement [8]. Possible strategies to tackle this issue have been described by experts in other countries. For example, a possible strategy to obtain updated information is including ARF and RHD as part of the diseases that require mandatory notification in each country [68,69]. Other more sophisticated strategies to identify contemporary data include the use of data linkage of multiple sources as employed by Katzenellenbogen et al. in Australia [70].

### 4.2. Burden of RHD

Data on the disease burden was the most identified in our review. An overall conclusion of the extracted information is that patients with RHD are heavily burdened by demise, the need for intervention (commonly reintervention), and disease complications particularly AFib, stroke, and HF. However, most publications were based on single-center studies that have inherent limitations as mentioned before. The Addis Ababa communiqué identified the lack of disease surveillance as one of the barriers to the situation of RHD in Africa [71]. The creation of prospective registers for an accurate depiction of the outcomes (morbidity and mortality) of RHD is a proposed solution to this barrier by the Addis Ababa communiqué, the WHF, and the American Heart Association [8,71,72]. Of the studies assessing burden, the experience of Nordet et al. in Cuba included the creation of a register of all the cases (5–25-year-olds) and assessing their outcomes, especially the need for admission, HF, and the need for valve surgery [48]. In the last decade, registers have been developed in other regions such as The Global Rheumatic Heart Disease Registry (REMEDY) and the VALVAFRIC [73,74]. The REMEDY study followed prospectively 3343 patients with RHD from African countries, India, and Yemen, identifying key information on the disease burden such as the severity of the disease and its complications such as HF, PH, AFib, and stroke [73]. On the other hand, the VALVAFRIC study was a retrospective registry that assessed patients with RHD in the hospital setting among eight countries in Western and Central Africa which described a compelling prevalence of in-hospital complications including HF, arrhythmias, IE, and EE as well as a 16% (94/1385) in-hospital mortality rate [74]. Registers aiming to collect information on the negative outcomes of RHD are missing in LAC, these efforts would be important to improve care and follow-up for patients with RHD [72].

Besides the high burden on the patients’ health, RHD also impacts the economy of each patient, their households, health systems, and governments [7]. Recently, a scoping review assessing the economic consequences of RHD, in which no studies from LAC fulfilled the eligibility criteria, concluded that most information on RHD costs comes from wealthier nations [75]. Consistent with this, our review only identified three studies from Brazil, Cuba, and Jamaica reflecting on the economic impact of RHD in the region [30,42,48]. In Africa, Coates et al. conducted a model of an investment case for RHD preventive and management strategies in the African Union from 2021 to 2030. They estimated that the total cost of increasing the coverage of all the interventions related to RHD during the ten-year period was US$3.9 billion [76]. Further investigations in LAC should include economic variables to assess the other side of the burden of RHD.

### 4.3. Prevention and screening of RHD

We identified six prevention programs, predominantly from study periods before 2000, that included a combination of strategies such as educational activities, secondary antibiotic prophylaxis, or the creation of registers. Three of them described a decline in many negative outcomes including the number of cases/recurrences, need for intervention, deaths, and costs [19,44,48]. For instance, the 10-year prevention program in Pinar del Rio, Cuba described a reduction in the incidence and severity of ARF/RHD as well as an increase in the compliance to secondary antibiotic prophylaxis [48]. A recent systematic review and meta-analysis described that prevention programs integrated (even if partially) into their country’s health system are beneficial in terms of health outcomes [77]. Moreover, echocardiographic screening programs such as the PROVAR study in Brazil, a large-scale program that focused initially on the echocardiographic screening of schoolchildren in Minas Gerais, have been successful in the early identification and consequent follow-up in patients with RHD [46]. Watkins et al. assessed the cost-effectiveness of the Pinar del Rio Program, a program that used a combination of primary and secondary preventive activities and identified that it was cost saving [78]. Similarly, a cost-effectiveness analysis of the PROVAR study deemed the strategy cost-effective in the Brazilian context [79]. Cost-effectiveness analyses are crucial for the implementation of these strategies by policymakers or health systems, especially in countries with limited resources as are many in LAC [78]. The modelling study by Coates et al. in the African Union described that investing in the prevention and management of RHD could markedly reduce the incidence and deaths due to RHD as well as provide net benefit of US$2.8 billion if coverage for secondary and tertiary care were scaled up [76]. The efforts for the prevention and early diagnosis of RHD in LAC require the involvement of different sectors, including the public health entities, the decision-makers, the scientific organizations, the clinicians, and the community.

### 4.4. Limitations

There are some limitations that are important to mention in our systematic review. First, we did not conduct additional searches for gray literature which could lead to missing information. However, we conducted a thorough search strategy, including two specific Latin American and Caribbean databases where journals from the region are indexed. Second, as we decided to include multiple study settings and methodologies, we could not provide an overall estimate or conclusion on the prevalence, incidence, or mortality of the disease as the studies varied widely in methodology and presented the data using distinct criteria, study periods, and study measures. The region requires updated population-based studies to further clarify the prevalence, incidence, morbidity, and mortality of these diseases. Third, in a similar trend as the previously mentioned limitation, we could not evaluate the trends or changes over time of epidemiological parameters such as prevalence/incidence or mortality due to the heterogeneity the lack of frequency data from multiple time periods among the included studies. This information is needed to assess the changes over time of the disease in LAC. Fourth, data were predominantly from a single nation, Brazil (65% of studies), which limits generalizability to the entire region, but this serves as an example for other countries in LAC to increase their research on RHD. This phenomenon could be explained due to a possible paucity of data or poor data quality in highly burdened regions and better data from regions or countries with better infrastructure [75,80]. Fifth, data on RHD-prevalence studies were mostly from the pediatric population and information was lacking on young adults and adults. This is an important aspect as according to the most recent GBD study, the peak age of RHD cases worldwide was 20–29 years [3].

## 5. Conclusions

This review summarizes the data from 48 primary studies in Latin America and the Caribbean. Most research and preventive efforts come from a single country, Brazil, thus identifying a need for other nations to ramp up their interest in this public health problem. The initial efforts should be aimed to develop up-to-date epidemiological studies, preferably population-based, or surveillance systems to have an accurate picture of RHD in each country and in the region. These data are crucial for the identification of possible areas, subregions, or countries that would require tailored strategies to reduce the heavy burden of RHD, such as the creation of register-based, echocardiographic screening, or comprehensive prevention programs.

## Data Accessibility Statement

All data is presented on the main text and supplementary materials (Supplementary Tables S1–S9).

## Additional File

The additional file for this article can be found as follows:

10.5334/gh.1152.s1 Supplementary Material. Supplementary Tables S1–S9 and PRISMA 2020 Checklist.

## References

[1] Carapetis JR, Beaton A, Cunningham MW, Guilherme L, Karthikeyan G, Mayosi BM, et al. Acute rheumatic fever and rheumatic heart disease. Nature Reviews Disease Primers. 2016; 2: 15084. DOI: 10.1038/nrdp.2015.84 PMC5810582 27188830

[2] Marijon E, Mocumbi A, Narayanan K, Jouven X, Celermajer DS. Persisting burden and challenges of rheumatic heart disease. European Heart Journal. 2021; 42: 3338–48. DOI: 10.1093/eurheartj/ehab407 34263296

[3] Roth GA, Mensah GA, Johnson CO, Addolorato G, Ammirati E, Baddour LM, et al. Global burden of cardiovascular diseases and risk factors, 1990–2019: Update from the GBD 2019 study. J Am Coll Cardiol. 2020; 76: 2982–3021. DOI: 10.1016/j.jacc.2020.11.010 33309175 PMC7755038

[4] Watkins DA, Johnson CO, Colquhoun SM, Karthikeyan G, Beaton A, Bukhman G, et al. Global, regional, and national burden of rheumatic heart disease, 1990–2015. New England Journal of Medicine. 2017; 377: 713–22. DOI: 10.1056/NEJMoa1603693 28834488

[5] Ordunez P, Martinez R, Soliz P, Giraldo G, Mujica OJ, Nordet P. Rheumatic heart disease burden, trends, and inequalities in the Americas, 1990–2017: A population-based study. The Lancet Global Health. 2019; 7: e1388–97. DOI: 10.1016/S2214-109X(19)30360-2 31537369

[6] Okello E, Wanzhu Z, Musoke C, Twalib A, Kakande B, Lwabi P, et al. Cardiovascular complications in newly diagnosed rheumatic heart disease patients at Mulago Hospital, Uganda. Cardiovascular Journal of Africa. 2013; 24: 80–5. DOI: 10.5830/CVJA-2013-004 23736132 PMC3721959

[7] Rwebembera J, Beaton AZ, de Loizaga SR, Rocha RTL, Doreen N, Ssinabulya I, et al. The global impact of rheumatic heart disease. Current Cardiology Reports. 2021; 23: 160. DOI: 10.1007/s11886-021-01592-2 34599389

[8] Remenyi B, Carapetis J, Wyber R, Taubert K, Mayosi BM. Position statement of the World Heart Federation on the prevention and control of rheumatic heart disease. Nature Reviews Cardiology. 2013; 10: 284–92. DOI: 10.1038/nrcardio.2013.34 23546444

[9] World Health Organization. Seventy-First World Health Assembly WHA71.14: Rheumatic Fever and Rheumatic Heart Disease; 2018.

[10] Kotit S, Phillips DIW, Afifi A, Yacoub M. The “Cairo Accord”- Towards the eradication of RHD: An update. Frontiers in Cardiovascular Medicine. 2021; 8: 690227. DOI: 10.3389/fcvm.2021.690227 34277735 PMC8282907

[11] Page MJ, McKenzie JE, Bossuyt PM, Boutron I, Hoffmann TC, Mulrow CD, et al. The PRISMA 2020 statement: An updated guideline for reporting systematic reviews. BMJ. 2021; 372: n71. DOI: 10.1136/bmj.n71 33782057 PMC8005924

[12] Jaimes-Reyes MA, Urina-Jassir M, Urina-Triana M, Urina-Triana M. Current situation of acute rheumatic fever and rheumatic heart disease in Latin America and the Caribbean: A systematic review. PROSPERO 2021 CRD42021250043 2021. https://www.crd.york.ac.uk/prospero/display_record.php?ID=CRD42021250043 (accessed March 31, 2022). 10.5334/gh.1152 PMC9438465 36199563

[13] Ouzzani M, Hammady H, Fedorowicz Z, Elmagarmid A. Rayyan-a web and mobile app for systematic reviews. Systematic Reviews. 2016; 5: 210. DOI: 10.1186/s13643-016-0384-4 27919275 PMC5139140

[14] Wells G, Shea B, O’Connell D, Peterson J, Welch V, Losos M, et al. The Newcastle-Ottawa Scale (NOS) for assessing the quality of nonrandomised studies in meta-analyses. The Ottawa Hospital Research Institute; 2021. http://www.ohri.ca/programs/clinical_epidemiology/oxford.asp (accessed April 18, 2021).

[15] Downes MJ, Brennan ML, Williams HC, Dean RS. Development of a critical appraisal tool to assess the quality of cross-sectional studies (AXIS). BMJ Open. 2016; 6: e011458. DOI: 10.1136/bmjopen-2016-011458 PMC5168618 27932337

[16] Sterne JAC, Savović J, Page MJ, Elbers RG, Blencowe NS, Boutron I, et al. RoB 2: A revised tool for assessing risk of bias in randomised trials. BMJ. 2019; l4898. DOI: 10.1136/bmj.l4898 31462531

[17] Eldridge S, Campbell MK, Campbell MJ, Drahota AK, Giraudeau B, Reeves BC, et al. Revised Cochrane risk of bias tool for randomized trials (RoB 2). Additional considerations for cluster-randomized trials (RoB 2 CRT); 2021. https://sites.google.com/site/riskofbiastool/welcome/rob-2-0-tool/rob-2-for-cluster-randomized-trials (accessed August 24, 2021).

[18] Alves Meira ZM, de Castilho SR, Lins Barros MV, Maria Vitarelli A, Diniz Capanema F, Moreira NS, et al. Prevalência da Febre Reumática em Crianças de uma Escola da Rede Pública de Belo Horizonte. Arq Bras Cardiol. 1995; 65: 331–4. 8728807

[19] Bach JF, Chalons S, Mosser A, Forier E, Elana G, Jouanelle J, et al. 10-year educational programme aimed at rheumatic fever in two French Caribbean islands. The Lancet. 1996; 347: 644–8. DOI: 10.1016/S0140-6736(96)91202-7 8596378

[20] Beaton A, Nascimento BR, Diamantino AC, Pereira GTR, Lopes ELV, Miri CO, et al. Efficacy of a standardized computer-based training curriculum to teach echocardiographic identification of rheumatic heart disease to nonexpert users. The American Journal of Cardiology. 2016; 117: 1783–9. DOI: 10.1016/j.amjcard.2016.03.006 27084054

[21] Berrios X, del Campo E, Guzman B, Bisno AL. Discontinuing rheumatic fever prophylaxis in selected adolescents and young adults: A prospective study. Annals of Internal Medicine. 1993; 118: 401–6. DOI: 10.7326/0003-4819-118-6-199303150-00001 8439112

[22] Canale LS, Colafranceschi AS, Monteiro AJO, Marques BM, Canale CS, Koehler EC, et al. Surgical treatment of atrial fibrillation using bipolar radiofrequency ablation in rheumatic mitral disease. Revista Brasileira de Cirurgia Cardiovascular. 2011; 26: 565–72. DOI: 10.5935/1678-9741.20110046 22358271

[23] Casalino R, Tarasoutchi F, Spina G, Katz M, Bacelar A, Sampaio R, et al. EuroSCORE models in a cohort of patients with valvular heart disease and a high prevalence of rheumatic fever submitted to surgical procedures. PLOS ONE. 2015; 10: e0118357. DOI: 10.1371/journal.pone.0118357 25714474 PMC4340937

[24] Chavez EK, Colafranceschi AS, Monteiro AJ de O, Canale LS, Mesquita ET, Weksler C, et al. Surgical treatment of atrial fibrillation in patients with rheumatic valve disease. Brazilian Journal of Cardiovascular Surgery. 2017; 32: 202–9. DOI: 10.21470/1678-9741-2017-0016 28832799 PMC5570393

[25] Cruz RCC, Cordeiro BS, Santos F de S, Fernandes CR, Gama JMA, Ladeia AMT. Predictors of unfavourable outcomes in children and adolescents submitted to surgical mitral valvuloplasty secondary to chronic rheumatic heart disease. Arquivos Brasileiros de Cardiología. 2019; 113: 748–56. DOI: 10.5935/abc.20190184 31508692 PMC7020859

[26] de Araújo Fonseca LG, Lima INDF, Gualdi LP. Characterization of Brazilian hospital admissions due to cardiovascular diseases: A longitudinal study. BMC Cardiovascular Disorders. 2020; 20: 311. DOI: 10.1186/s12872-020-01588-w 32600334 PMC7325147

[27] Defilló Ricart M, López Mateo M, Garrido Contreras E, Contreras Graves P, Batista Batista D, Fernández Estrada J. Fiebre reumatica en niños menores de 5 años: Revisión clínica de 20 años (1969–1989) Hospital Infantil Dr. Robert Reid Cabral, Santo Domingo. Acta Medica Dominicana. 1991; 13: 1–6.

[28] Durães AR, Durães MAO, Correia LC, Fernandes AMS, Aras Júnior R. Impact of aspirin use in the incidence of thromboembolic events after bioprosthesis replacement in patients with rheumatic disease. Revista Brasileira de Cirurgia Cardiovascular. 2013; 28: 347–52. DOI: 10.5935/1678-9741.20130054 24343684

[29] Fernandes AMS, Oliveira RM, de Andrade GM, Biscaia GT, Medrado Junior FA, dos Reis FFB, et al. In-hospital mortality in patients with rheumatic heart disease undergoing double valve replacement. International Journal of Cardiovascular Sciences. 2015; 28: 298–304. DOI: 10.5935/2359-4802.20150043

[30] Figueiredo ET de, Azevedo L, Rezende ML, Alves CG. Rheumatic fever: A disease without color. Arquivos Brasileiros de Cardiología. 2019; 113: 345–54. DOI: 10.5935/abc.20190141 31365604 PMC6882402

[31] Giachetto G, Franco S, Guariglia R. Aspectos epidemiológicos y clínicos de la fiebre reumática: Centro Hospitalario Pereira Rossell 1990–1993. Arch Pediatr Uruguay. 1994; 65: 11–5.

[32] Haddad N, Bittar OJNV. Diagnósticos mais freqüentes em pacientes internados em hospital especializado em cardiologia: evolução em 15 anos (1988 a 2003). Rev adm saúde. 2005; 7: 7–11.

[33] Haddad N, Silva MB da. Mortality due to cardiovascular disease in women during the reproductive age (15 to 49 years), in the state of São Paulo, Brazil, from 1991 to 1995. Arquivos Brasileiros de Cardiología. 2000; 75: 375–9. DOI: 10.1590/S0066-782X2000001100002 11080749

[34] Isaacura C, Granero R. Tendencia en la mortalidad por fiebre reumática aguda y cardiopatía reumática crónica en Venezuela, 1955–1994. Cadernos de Saúde Pública. 1998; 14: 165–9. DOI: 10.1590/S0102-311X1998000100024 9592222

[35] Jatene MB, Marcial MB, Tarasoutchi F, Cardoso RA, Pomerantzeff P, Jatene AD. Influence of the maze procedure on the treatment of rheumatic atrial fibrillation – Evaluation of rhythm control and clinical outcome in a comparative study. European Journal of Cardio-Thoracic Surgery. 2000; 17: 117–24. DOI: 10.1016/S1010-7940(00)00326-2 10731646

[36] Lavitola P de L, Sampaio RO, Oliveira WA de, Bôer BN, Tarasoutchi F, Spina GS, et al. Warfarin or aspirin in embolism prevention in patients with mitral valvulopathy and atrial fibrillation. Arq Bras Cardiol. 2010; 95: 749–55. DOI: 10.1590/S0066-782X2010005000140 20976374

[37] Lolio CA de, Laurenti R, Buchala CM, Santo AH, Mello Jorge MHP de. Mortalidade de mulheres em idade fértil no Município de São Paulo (Brasil), 1986: III- Mortes por diferentes causas: doenças cardiovasculares. Revista de Saúde Pública. 1991; 25: 37–40. DOI: 10.1590/S0034-89101991000100008 1784960

[38] Luque C, Cisternas FA, Araya M. Cambios del patrón de enfermedad en la postransición epidemiológica en salud en Chile, 1950–2003. Revista Médica de Chile. 2006; 134: 703–12. DOI: 10.4067/S0034-98872006000600005 17130944

[39] Meira ZMA, Goulart EMA, Colosimo EA, Mota CCC. Long-term follow-up of rheumatic fever and predictors of severe rheumatic valvar disease in Brazilian children and adolescents. Heart. 2005; 91: 1019–22. DOI: 10.1136/hrt.2004.042762 16020588 PMC1769032

[40] Mejia OAV, Antunes MJ, Goncharov M, Dallan LRP, Veronese E, Lapenna GA, et al. Predictive performance of six mortality risk scores and the development of a novel model in a prospective cohort of patients undergoing valve surgery secondary to rheumatic fever. PLOS ONE. 2018; 13: e0199277. DOI: 10.1371/journal.pone.0199277 29979692 PMC6034795

[41] Meneguz-Moreno RA, Costa JR, Gomes NL, Braga SLN, Ramos AIO, Meneghelo Z, et al. Very long-term follow-up after percutaneous balloon mitral valvuloplasty. JACC: Cardiovascular Interventions. 2018; 11: 1945–52. DOI: 10.1016/j.jcin.2018.05.039 30077684

[42] Millard-Bullock D. The rheumatic fever and rheumatic heart disease control programme – Jamaica. West Indian Medical Journal. 2012; 61: 361–4. DOI: 10.7727/wimj.2012.134 23240469

[43] Miranda LP, Camargos PAM, Torres RM, Meira ZMA. Prevalence of rheumatic heart disease in a public school of Belo Horizonte. Arq Bras Cardiol. 2014; 103: 89–97. DOI: 10.5935/abc.20140116 25211312 PMC4150659

[44] Mota CCC, Meira ZMA, Graciano RN, Graciano FF, Araújo FDR. Rheumatic fever prevention program: long-term evolution and outcomes. Front Pediatr. 2015; 2: 141. DOI: 10.3389/fped.2014.00141 25610826 PMC4285057

[45] Nascimento BR, Sable C, Nunes MCP, Oliveira KKB, Franco J, Barbosa MM, et al. Echocardiographic screening of pregnant women by non-physicians with remote interpretation in primary care. Family Practice. 2021; 38: 225–30. DOI: 10.1093/fampra/cmaa115 33073294

[46] Nascimento BR, Sable C, Nunes MCP, Diamantino AC, Oliveira KKB, Oliveira CM, et al. Comparison between different strategies of rheumatic heart disease echocardiographic screening in Brazil: Data from the PROVAR (Rheumatic Valve Disease Screening Program) Study. J Am Heart Assoc. 2018; 7: e008039. DOI: 10.1161/JAHA.117.008039 29444774 PMC5850205

[47] Noah PK. Trends in acute rheumatic fever. The Barbados experience. J Trop Pediatr. 1994; 40: 94–6. DOI: 10.1093/tropej/40.2.94 8015038

[48] Nordet P, Lopez R, Dueñas A, Sarmiento L. Prevention and control of rheumatic fever and rheumatic heart disease: The Cuban experience (1986–1996–2002). Cardiovascular Journal of Africa. 2008; 19: 135–40. 18568172 PMC3974561

[49] Nunes MCP, Hung J, Barbosa MM, Esteves WA, Carvalho VT, Lodi-Junqueira L, et al. Impact of net atrioventricular compliance on clinical outcome in mitral stenosis. Circulation: Cardiovascular Imaging. 2013; 6: 1001–8. DOI: 10.1161/CIRCIMAGING.112.000328 24097419 PMC3896378

[50] Oliveira KKB, Nascimento BR, Beaton AZ, Nunes MCP, Silva JLP, Rabelo LC, et al. Health education about rheumatic heart disease: A community-based cluster randomized trial: Rheumatic heart disease educational strategies. Global Heart. 2020; 15: 41. DOI: 10.5334/gh.347 32923335 PMC7413209

[51] Paar JA, Berrios NM, Rose JD, Cáceres M, Peña R, Pérez W, et al. Prevalence of rheumatic heart disease in children and young adults in Nicaragua. The American Journal of Cardiology. 2010; 105: 1809–14. DOI: 10.1016/j.amjcard.2010.01.364 20538135 PMC2895982

[52] Pato MF, Gelape CL, Cassiano TJ, Carvalho A, Cintra PR, Passaglia LG, et al. Determinants of prolonged length of hospital stay after cardiac surgery: Impact of rheumatic heart disease. Medical Express (Sao Paulo, Online). 2015; 2: M150304. DOI: 10.5935/MedicalExpress.2015.03.04

[53] Ribeiro GS, Tartof SY, Oliveira DWS, Guedes ACS, Reis MG, Riley LW, et al. Surgery for valvular heart disease: A population-based study in a Brazilian urban center. PLoS ONE. 2012; 7: e37855. DOI: 10.1371/journal.pone.0037855 22666401 PMC3362603

[54] Salinas Mondragón C, Lapoint Montes M. Demanda hospitalaria, manifestaciones clínicas y prevención de fiebre reumática y cardiopatía reumática en el Instituto de Salud del Niño 1989–1993. Diagnóstico (Perú). 1995; 34: 15–24.

[55] Seguel E, Alarcón E, González R, Stockins A, Neira S. L. Reemplazo valvular mitral en valvulopatía reumática: ¿es beneficioso conservar el velo posterior? Revista Chilena de Cardiología. 2008; 27: 460–9.

[56] Severino ESB de O, Petrucci O, Vilarinho KA de S, Lavagnoli CFR, Silveira Filho L da M, Oliveira PPM de, et al. Late outcomes of mitral repair in rheumatic patients. Revista Brasileira de Cirurgia Cardiovascular. 2011; 26: 559–64. DOI: 10.5935/1678-9741.20110045 22358270

[57] Silva AP, Silva ML, Silva DB da. Frequência de internações por febre reumática em um hospital pediátrico de referência em um período de 20 anos. Revista Paulista de Pediatria. 2010; 28: 141–7. DOI: 10.1590/S0103-05822010000200003

[58] Soto López ME, Cordera González de Cosío F, Estrada L, Guel L, Abud Mendoza C, Reyes PA. Fiebre reumática en el quinquenio 1994–1999 en dos hospitales en San Luis Potosí y en México D.F. Archivos de Cardiologia de Mexico. 2001; 71: 127–35. 11565304

[59] Souza MJ de, Benchetrit LC, Oliveira A de. Estreptococos beta-hemolíticos e doença reumática cardíaca em crianças no Rio de Janeiro, Brasil. Folha méd. 1990; 101: 79–92.

[60] Spitzer E, Mercado J, Islas F, Rothenbühler M, Kurmann R, Zürcher F, et al. Screening for rheumatic heart disease among Peruvian children: A two-stage sampling observational study. PLOS ONE. 2015; 10: e0133004. DOI: 10.1371/journal.pone.0133004 26208006 PMC4514892

[61] Stokes Baltazar WR. Diagnóstico de fiebre reumática en una clínica privada de Izabal, Departamento de Guatemala. Revista de Medicina Interna de Guatemala. 2007; 16: 19–29.

[62] Travancas PR, Dorigo AH, Simões LC, Fonseca SC, Bloch K v, Herdy G v. Comparison of mechanical and biological prostheses when used to replace heart valves in children and adolescents with rheumatic fever. Cardiol Young. 2009; 19: 192–7. DOI: 10.1017/S1047951109003680 19267944

[63] Vasconcelos M, Vasconcelos L, Ribeiro V, Campos C, Di-Flora F, Abreu L, et al. Incidence and predictors of stroke in patients with rheumatic heart disease. Heart. 2021; 107: 748–54. DOI: 10.1136/heartjnl-2020-318054 33414162

[64] WHO Cardiovascular Diseases Unit and principal investigators. WHO programme for the prevention of rheumatic fever/rheumatic heart disease in 16 developing countries: Report from Phase I (1986–90). Bull World Health Organ. 1992; 70: 213–8. 1600581 PMC2393294

[65] Zabal C, Attie F, Barragán R, Buendía A, Peña Duque M. Resultado tardio del cambio valvular mitral en 155 sujetos menores de 16 años. Estudio comparativo con cuatro protesis. Archivos del Instituto de Cardiologia de Mexico. 1992; 62: 333–8. 1417351

[66] Noubiap JJ, Agbor VN, Bigna JJ, Kaze AD, Nyaga UF, Mayosi BM. Prevalence and progression of rheumatic heart disease: A global systematic review and meta-analysis of population-based echocardiographic studies. Scientific Reports. 2019; 9: 17022. DOI: 10.1038/s41598-019-53540-4 31745178 PMC6863880

[67] Beaton A, Okello E, Rwebembera J, Grobler A, Engelman D, Alepere J, et al. Secondary antibiotic prophylaxis for latent rheumatic heart disease. New England Journal of Medicine. 2022; 386: 230–40. DOI: 10.1056/NEJMoa2102074 34767321

[68] Negi PC, Sondhi S, Asotra S, Mahajan K, Mehta A. Current status of rheumatic heart disease in India. Indian Heart Journal. 2019; 71: 85–90. DOI: 10.1016/j.ihj.2018.12.007 31000189 PMC6477130

[69] Zühlke LJ, Engel ME, Watkins D, Mayosi BM. Incidence, prevalence and outcome of rheumatic heart disease in South Africa: A systematic review of contemporary studies. International Journal of Cardiology. 2015; 199: 375–83. DOI: 10.1016/j.ijcard.2015.06.145 26247792

[70] Katzenellenbogen JM, Bond-Smith D, Seth RJ, Dempsey K, Cannon J, Stacey I, et al. Contemporary incidence and prevalence of rheumatic fever and rheumatic heart disease in Australia using linked data: The case for policy change. J Am Heart Assoc. 2020; 9: e016851. DOI: 10.1161/JAHA.120.016851 32924748 PMC7792417

[71] Watkins D, Zuhlke L, Engel M, Daniels R, Francis V, Shaboodien G, et al. Seven key actions to eradicate rheumatic heart disease in Africa: The Addis Ababa communiqué. Cardiovascular Journal of Africa. 2016; 27: 184–7. DOI: 10.5830/CVJA-2015-090 26815006 PMC5125265

[72] Kumar RK, Antunes MJ, Beaton A, Mirabel M, Nkomo VT, Okello E, et al. Contemporary diagnosis and management of rheumatic heart disease: Implications for closing the gap: A scientific statement from the American Heart Association. Circulation. 2020; 142: e337–57. DOI: 10.1161/CIR.0000000000000921 33073615

[73] Zühlke L, Engel ME, Karthikeyan G, Rangarajan S, Mackie P, Cupido B, et al. Characteristics, complications, and gaps in evidence-based interventions in rheumatic heart disease: The Global Rheumatic Heart Disease Registry (the REMEDY study). European Heart Journal. 2015; 36: 1115–22. DOI: 10.1093/eurheartj/ehu449 25425448 PMC4422972

[74] Kingué S, Ba SA, Balde D, Diarra MB, Anzouan-Kacou JB, Anisubia B, et al. The VALVAFRIC study: A registry of rheumatic heart disease in Western and Central Africa. Archives of Cardiovascular Diseases. 2016; 109: 321–9. DOI: 10.1016/j.acvd.2015.12.004 26988837

[75] Opara CC, Aghassibake N, Watkins DA. Economic consequences of rheumatic heart disease: A scoping review. International Journal of Cardiology. 2021; 323: 235–41. DOI: 10.1016/j.ijcard.2020.09.020 32920073

[76] Coates MM, Sliwa K, Watkins DA, Zühlke L, Perel P, Berteletti F, et al. An investment case for the prevention and management of rheumatic heart disease in the African Union 2021–30: A modelling study. The Lancet Global Health. 2021; 9: e957–66. DOI: 10.1016/S2214-109X(21)00199-6 33984296 PMC9087136

[77] Abrams J, Watkins DA, Abdullahi LH, Zühlke LJ, Engel ME. Integrating the prevention and control of rheumatic heart disease into country health systems: A systematic review and meta-analysis. Global Heart. 2020; 15: 62. DOI: 10.5334/gh.874 33150127 PMC7500229

[78] Watkins DA, Mvundura M, Nordet P, Mayosi BM. A cost-effectiveness analysis of a program to control rheumatic fever and rheumatic heart disease in Pinar del Rio, Cuba. PLOS ONE. 2015; 10: e0121363. DOI: 10.1371/journal.pone.0121363 25768008 PMC4358951

[79] Ubels J, Sable C, Beaton AZ, Nunes MCP, Oliveira KKB, Rabelo LC, et al. Cost-effectiveness of rheumatic heart disease echocardiographic screening in Brazil: Data from the PROVAR+ study: Cost-effectiveness of RHD screening in Brazil. Glob Heart. 2020; 15: 18. DOI: 10.5334/gh.529 32489791 PMC7218764

[80] Zühlke LJ, Steer AC. Estimates of the global burden of rheumatic heart disease. Global Heart. 2013; 8: 189–95. DOI: 10.1016/j.gheart.2013.08.008 25690495

