# Supplementary material for: Current Situation of Acute Rheumatic Fever and Rheumatic Heart Disease in Latin America and the Caribbean: A Systematic Review
Source: Glob Heart. 2022 Sep 2;17(1):65. doi: 10.5334/gh.1152 (PMC9438465; doi:10.5334/gh.1152)
Supplement: Supplementary Material. — Supplementary Tables S1–S9 and PRISMA 2020 Checklist. [file gh-17-1-1152-s1.pdf]

Supplementary Material

1. **Supplementary table S1 – S4. Search strings for each database** Pages 2 – 3
2. **Supplementary table S5. Characteristics of included studies** Pages 4 – 11
3. **Supplementary table S6 – S8. Quality assessment tools per included studies** Pages 11 – 13
4. **Supplementary table S9. Burden of rheumatic heart disease per included studies.** Pages 14 – 17
5. **PRISMA 2020 Checklist** Pages 18 – 20

Supplementary table S1. Search Strategy for LILACS database, Date of the Last Search: April 22nd, 2021

#	Query	Total
**1**	(“Rheumatic Fever”) OR (“Fiebre Reumatica”) OR (“Rheumatic Heart Disease”) OR (“Cardiopatia Reumatica”)	960
**2**	**Date Filter:** 1990 - 2021	734

Supplementary table S2. Search Strategy for SciELO database, Date of the Last Search: April 22nd, 2021

#	Query	Total
**1**	(“Rheumatic Fever”) OR (“Fiebre Reumatica”) OR (“Rheumatic Heart Disease”) OR (“Cardiopatia Reumatica”)	279
**2**	**Date Filter:** 1990 - 2021	261

Supplementary table S3. Search Strategy for PubMed database, Date of the Last Search: April 22nd, 2021

#	Query	Total
**1**	"Rheumatic Fever"[Mesh] OR "Rheumatic Heart Disease"[Mesh] OR "Rheumatic Fever" OR "Rheumatic Heart Disease"	24,760
**2**	"Caribbean Region"[Mesh] OR "Latin America"[Mesh] OR "South America"[Mesh] OR "Central America"[Mesh] OR (“Americas”[Mesh] NOT “North America”[Mesh]) OR "Mexico"[Mesh] OR “Antigua and Barbuda” OR “Argentina” OR “Aruba” OR “Bahamas” OR “Barbados” OR “Belize” OR “Bolivia” OR “Brazil” OR “Virgin Islands” OR “Cayman Islands” OR “Chile” OR “Colombia” OR “Costa Rica” OR “Cuba” OR “Curacao” OR “Dominica” OR “Dominican Republic” OR “Ecuador” OR “El Salvador” OR “French Guiana” OR “Grenada” OR “Guadeloupe” OR “Guatemala” OR “Guyana” OR “Haiti” OR “Honduras” OR “Jamaica” OR “Martinique” OR “Mexico” OR “Nicaragua” OR “Panama” OR “Paraguay” OR “Peru” OR “Puerto Rico” OR “Saint Barthelemy” OR “St. Martin” OR “St. Kitts and Nevis” OR “St. Lucia” OR “St. Vincent and the Grenadines” OR “Suriname” OR “Trinidad and Tobago” OR “Turks and Caicos Islands” OR “Uruguay” OR “Venezuela”	907,984
**3**	**#1 AND #2**	643
**4**	**Date Filter Applied: 1990/1/1 - 2021/12/31**	494

Supplementary table S4. Search Strategy for EMBASE database, Date of the Last Search: April 22nd, 2021

#	Query	Total

**1**	'rheumatic fever'/exp OR 'rheumatic heart disease'/exp	28,012
**2**	'South and Central America'/exp OR 'Caribbean'/exp OR ('Americas' NOT 'North America') OR 'Mexico'/exp OR ‘Antigua and Barbuda’ OR ‘Argentina’ OR ‘Aruba’ OR ‘Bahamas’ OR ‘Barbados’ OR ‘Belize’ OR ‘Bolivia’ OR ‘Brazil’ OR ‘Virgin Islands’ OR ‘Cayman Islands’ OR ‘Chile’ OR ‘Colombia’ OR ‘Costa Rica’ OR ‘Cuba’ OR ‘Curacao’ OR ‘Dominica’ OR ‘Dominican Republic’ OR ‘Ecuador’ OR ‘El Salvador’ OR ‘French Guiana’ OR ‘Grenada’ OR ‘Guadeloupe’ OR ‘Guatemala’ OR ‘Guyana’ OR ‘Haiti’ OR ‘Honduras’ OR ‘Jamaica’ OR ‘Martinique’ OR ‘Mexico’ OR ‘Nicaragua’ OR ‘Panama’ OR ‘Paraguay’ OR ‘Peru’ OR ‘Puerto Rico’ OR ‘Saint Barthelemy’ OR ‘St. Martin’ OR ‘St. Kitts and Nevis’ OR ‘St. Lucia’ OR ‘St. Vincent and the Grenadines’ OR ‘Suriname’ OR ‘Trinidad and Tobago’ OR ‘Turks and Caicos Islands’ OR ‘Uruguay’ OR ‘Venezuela’	1,536,103
**3**	**#1 AND #2**	1,515
**4**	**Years published Filter Applied: 1990 - 2021**	942

Supplementary Table S5. Characteristics of included studies

Reference	Country (ies)	Study design	Objective	Study period	Setting	Topic			Total n	Population characteristics
						**Epi.**	**Bur.**	**Prev./ Scree.**		
Alves Meira et al., 1995 [18]	Brazil	Cross-sectional	"To determine the prevalence of RF among children of a public high school in Belo Horizonte, Brazil."	March - December 1992	School	**X**			550	Age range: 10 – 20 years<br>Female: 319 (58%)<br>Male 231 (42%)
Bach et al., 1996 [19]	Martinique and Guadeloupe	Cross-sectional	"To report how the Martinique/Guadeloupe eradication program was set up and results over 10 years"	1982 - 1992	School, hospital	**X**		**X**	N/A	N/A
Beaton et al., 2016[20]<br><br>***PROVAR study***	Brazil	Cross-sectional	"To test the ability of nonexperts with a variety of echocardiography experience to interpret RHD screening echocardiograms using handheld echocardiography after a brief, asynchronous, standardized, computer-based training course on image interpretation."	N/A	School			**X**	N/A	N/A
Berrios et al., 1993 [21]	Chile	Cohort	"To assess the safety of discontinuing prophylaxis with antimicrobial agents in patients judged to be at relatively low risk for recurrence of ARF"	July 1982 - September 1988	Community	**X**		**X**	59	Age range: 15 – 44<br>Mean age: 24.5<br>Female: 40 (67.8%)<br>Male: 19 (32.2%)
Canale et al., 2011[22]	Brazil	Cohort	"To report the mid-term clinical results and heart rate of patients undergoing bipolar radiofrequency ablation concomitant with mitral valve surgery of rheumatic origin at National Institute of Cardiology, Rio de Janeiro"	January 2008 - December 2009	Hospital (SC)		**X**		53	Surgical population<br>Mean age ± SD: 49.3 ± 10.7<br>Female: 34 (64%)<br>Male: 19 (36%)
Casalino et al., 2015[23]	Brazil	Cross-sectional	"To evaluate the performance of additive, logistic, and EuroSCORE II in predicting in-hospital mortality in a cohort of patients with valvular heart disease and a predominant rheumatic etiology submitted to surgical procedure in a tertiary	February - December 2009	Hospital (SC)		**X**		247 with RHD (Total: 440)	Surgical population<br>Mean age ± SD: 51 ± 12<br>Female: 150 (60.3%)

			hospital that receives patients from all over Brazil"							
Chavez et al., 2017[24]	Brazil	Cohort	"To evaluate of the cardiac rate at hospital discharge and a one-year postoperative follow-up period as well as factors associated with the occurrence of sinus rhythm at the end of the first year with patients presenting RHD and indication for mitral valve surgery concomitant with surgical ablation of AF"	January 2013 - December 2014	Hospital (SC)		**X**		103	Surgical population<br>Mean age ± SD: 50.76 ± 10.7<br>Female: 78 (76%)<br>Male: 25 (24%)
Cruz et al., 2019[25]	Brazil	Cohort	"To identify predictors of unfavorable outcome in children and adolescents submitted to mitral valvuloplasty secondary to RHD"	March 2011 - January 2017	Hospital (SC)		**X**		54	Surgical Population<br>Mean age ± SD: 10.5 ±3.2<br>Female: 29 (53.7%)<br>Male: 25 (46.3%)
de Araújo Fonseca et al., 2020 [26]	Brazil	Cross-sectional	"To characterize Brazilian hospital admissions distribution classified by the ICD-10 in adults between 2008 and 2017, as well as to analyze the incidence of hospital admissions, the regional distribution, and to observe the temporal trend of hospital admissions and mortality rate due to CVD in Brazil."	2008 – 2017	Hospital nationwide (MC)	**X**			11,345,821<br><br>**(***n* of CVD admissions)	N/A
Defilló Ricart et al., 1991 [27]	Dominican Republic	Cross-sectional	To establish the number of ARF cases and their clinical characteristics throughout 20 years	November 1969 - December 1989	Hospital (SC)	**X**			19,483 (*n* of admissions)	N/A
Durães et al., 2013 [28]	Brazil	Cohort	"To determine the contemporary incidence of thromboembolic events in rheumatic patients early after isolated aortic and mitral bio prosthesis replacement and perform a comparison between isolated use of aspirin with no-antiplatelet therapy"	January 2010 - July 2012	Hospital (SC)		**X**		184	Surgical population<br>Mean age ± SD: 45 ± 16 (aspirin group); 42 ± 15 (no intervention group)<br>Female: 94 (51%)<br>Male: 90 (49%)
Fernandes et al., 2015 [29]	Brazil	Cross-sectional	"To identify potential associations between surgical variables and in-hospital mortality of patients with RHD who have undergone DVR."	January 2007 - December 2011	Hospital (SC)		**X**		104	Surgical population<br>Mean age ± SD: 38.04 ± 14.4<br>Female: 44 (42.3%)<br>Male: 60 (57.7%)
Figueiredo et al., 2019 [30]	Brazil	Cross-sectional	"To analyze the historical series of mortality rates and disease costs, projecting future trends to offer new data that may justify the need to implement a public health program for ARF."	1998 – 2016	Population		**X**		N/A	N/A
Giachetto et al., 1994 [31]	Uruguay	Cross-sectional	"To analyze the clinic and epidemiologic aspects of ARF at	January 1990 - December 1993	Hospital (SC)	**X**			8,082 (*n* of admissions)	*Characteristics of ARF cases (n=58)

			the Centro hospitalario Pereira Rossell between 1990 -1993"							Age range: 2-14<br>Female: 24 (41.4%)<br>Male: 34 (58.6%)
Haddad and Silva, 2000 [33]	Brazil	Cross-sectional	"To describe mortality due to CVD in women during the reproductive age (15 to 49 years) in the state of São Paulo, Brazil, from 1991 to 1995"	1991 – 1995	Population		**X**		71,041 deaths – 22.8% CVD deaths	Age range: 15 – 49<br>Female: 100%
Haddad and Bittar, 2005 [32]	Brazil	Cross-sectional	"To assess the most frequent diagnosis of patients admitted to a hospital specializing in CVD and their evolution in a 15-year period, from June 1988 to June 2003"	June 1988 - June 2003	Hospital (SC)	**X**			N/A	N/A
Isaacura and Granero, 1998 [34]	Venezuela	Cross-sectional	"To present the mortality tendency of ARF and RHD in Venezuela from 1955 - 1994"	1955 – 1994	Population		**X**		N/A	N/A
Jatene et al., 2000 [35]	Brazil	Cohort	"To evaluate the efficacy of the Cox maze procedure in the treatment of rheumatic AF associated with surgical treatment of the mitral valve in consecutive patients."	July 1991 - June 1994	Hospital (SC)		**X**		55	Mean age: 51.5<br>Female: 47 (85.5%)<br>Male: 8 (14.5%)
Lavitola et al., 2010[36]	Brazil	RCT	"To evaluate whether the aspirin therapy can be used in patients with RMVD and AF as an effective alternative to warfarin, in a population at risk for thromboembolic events presenting treatment adherence difficulties when submitted to warfarin therapy."	N/A	Hospital (SC)		**X**		229	Adult population only<br>Female: 180 (78.6%)<br>Male: 49 (21.4%)
Lolio et al., 1991[37]	Brazil	Cross-sectional	"To present the general results on deaths by age, gender, and cause, as well as to study deaths for CVD"	July - December 1986	Population		**X**		953 deaths – 23.6% CVD deaths	Age range: 10-49<br>Female: 100%
Luque et al., 2006[38]	Chile	Cross-sectional	"To evaluate the epidemiological changes of allergic, autoimmune, and infectious diseases in Chile between 1950 and 2003."	1978 – 1998	Population	**X**			N/A	N/A
Meira et al., 2005[39]	Brazil	Cohort	"To study the progress of valvar disease by means of clinical and echocardiographic evaluations and to identify the independent variables that predict severe chronic valvar disease"	August 1983 - December 1998	Hospital (SC)	**X**			258	Mean age ± SD at first ARF attack: 9 ± 2.6
Mejia et al., 2018 [40]	Brazil	Cohort	"To evaluate the predictive performance of six different risk scores: the 2000 Bernstein-Parsonnet, EuroSCORE II,	May 2010 - July 2015	Hospital (SC)		**X**		2919	Surgical population<br>Mean age ± SD:<br>- Survived: 51.2 ± 14.9<br>- Death: 54.4 ± 17.4

			InsCor, Ambler, Guaragna and the New York scores."							
Meneguz-Moreno et al., 2018 [41]	Brazil	Cohort	"To assess very long-term outcomes after successful PBMV"	August 1987 - July 2010	Hospital (SC)		**X**		1582	Surgical population<br>Mean age ± SD: 36.44 ± 12.77<br>Female: 1362 (86.1%)<br>Male: 220 (13.9%)
Millard-Bullock, 2012 [42]	Jamaica	Cross-sectional	To present the experience of the ARF and RHD control program in Jamaica	1975-1985, 1989-1995	Hospital (MC)	**X**		**X**	Survey A: 1079<br>Survey B: 512	N/A
Miranda et al., 2014 [43]	Brazil	Cross-sectional	"To assess the prevalence of RHD according to physical examination and Doppler echocardiography in students at a public school of Belo Horizonte"	May 2010 - November 2011	School	**X**			267	Age range: 6-16<br>Female: 140 (52.4%)<br>Male: 127 (47.6%)
Mota et al., 2015[44]	Brazil	Cohort	"To analyze the profile of long-term evolution of patients with rheumatic valvar lesions and the outcomes after the control of recurrences, which followed the implementation of the Prevention Program for RF-UFMG"	July 1977 - February 2000	Hospital (SC)			**X**	702	Mean age ± SD at admission: 10.3 ± 3.1<br>Female: 50.4%<br>Male: 49.6%
Nascimento et al., 2018 [46]<br><br>***PROVAR study***	Brazil	Cross-sectional	"To evaluate the impact of these measures by comparing the participation rate and prevalence and pattern of latent RHD between public schools, private schools, and primary care centers in school-aged children."	October 2014 - December 2016	School, primary care centers	**X**		**X**	12048	Median age (IQR): 13 (11-15)<br>Female: 6675 (55.4%)<br>Male: 5373 (44.6%)
Nascimento et al., 2021 [45]<br><br>***PROVAR + study***	Brazil	Cross-sectional	"To evaluate the feasibility of integrating the PROVAR+ strategy into Brazilian prenatal PC, and to assess the prevalence of pre-existing HD in this population"	January 2018 - January 2019	Community	**X**		**X**	1112	Pregnant only<br>Mean age ± SD: 26.8 ± 7.5
Noah, 1994 [47]	Barbados	Cross-sectional	"To determine the existing trend in the incidence of ARF in Barbados"	January 1971 - December 1990	Population	**X**			258100 (Census 1991)<br><br>56057 (School population)<br><br>66829 (Childhood < 19 y)	N/A
Nordet et al., 2008[48]	Cuba	Cross-sectional	"To report how the Pinar del Rio project was planned and implemented, and present the results of the 15-year plan"	January 1986 - December 2001	School (Prevalence)<br><br>Population (Incidence, Burden)	**X**	**X**	**X**	<u>Prevalence</u><br>1985 (5-14-year-old): 5708<br>1996 (5-14-year-old): 25519<br><br><u>Incidence</u>:<br>1986 (5-25-year-old): 279400	N/A

									1996 (5-25-year-old): 233898<br>2002 (5-25-year-old): 207815<br>1986 (5-14-year-old): 119600<br>1996 (5-14-year-old): 106302<br>2002 (5-14-year-old): 104375<br><br><u>Burden</u><br>1986-90 (5-25-year-old): 134<br>1991-96 (5-25-year-old): 193	
Nunes et al., 2013[49]	Brazil	Cohort	"To determine the incremental prognostic value of Cn and its independent contribution to pulmonary artery pressure in a substantial population of patients with MS."	2007 - 2011	Hospital (SC)		**X**		128	Mean age ± SD: 42.6 ± 11.2<br>Female: 116 (90.6%)<br>Male: 12 (9.4%)
Oliveira et al., 2020[50]	Brazil	Cluster randomized trial	"To evaluate the effectiveness of transmission and retention of knowledge resulting from an educational process on pharyngitis, ARF and RHD in Brazilian public schools, comparing the results of two teaching methods"	2016 - 2017	School			**X**	1301 (90 clusters)	Mean age ± SD:<br>- Group 1: 15.2 ± 1.9<br>- Group 2: 15.1 ± 2.0<br>Female: 677 (52%)<br>Male: 624 (48%)
Paar et al., 2010[51]	Nicaragua	Cross-sectional	"To establish the true prevalence of RHD in the population using physical examinations and Doppler echo criteria"	2006 - 2009	Community	**X**			Pediatric: 3150<br>Adults: 489	Pediatric<br>- Mean age: 9.5<br>- Female: 1531 (49%)<br>- Male: 1619 (51%)<br><br>Adult:<br>- Mean age: 26.8<br>- Female: 305 (62%)<br>- Male: 184 (38%)
Pato et al., 2015[52]	Brazil	Cross-sectional	"To determine the additional value of RHD in predicting morbidity after cardiac valve surgery in the current era of heart valve disease treatment"	June 2010 - June 2011	Hospital (SC)		**X**		32 with RHD (total n: 164)	Surgical population<br>Mean age ± SD: 45.9 ± 14.0<br>Female: 22 (69%)<br>Male: 10 (31%)
Ribeiro et al., 2012 [53]	Brazil	Cross-sectional	"To determine the incidence and the most common etiologies for valvular heart dysfunctions requiring surgical intervention in Salvador, Brazil."	January 2002 - December 2005	Population		**X**		296 with RHD (total n: 491)	Surgical population<br>Median age: 37 (25 - 48)<br>Female: 167 (56%)<br>Male: 129 (44%)

Salinas Mondragón et al., 1995 [54]	Peru	Cross-sectional	"To report the results of our research on ARF/RHD and prove that ARF/RHD is still a health problem for children and adults in the country"	1989 - 1993	Hospital (SC)	**X**	**X**		1074 discharges<br>66 due to ARF/RHD (**Burden**)	N/A
Seguel et al., 2008 [55]	Chile	Cohort	"To evaluate results of MVR with preservation of the posterior leaflet in patients with RHD"	1990 - 2004	Hospital (SC)		**X**		212	Surgical population<br>Mean age:<br>- Veil preservation: 45.53<br>- No preservation: 43.66<br>Female: 168 (79.2%)<br>Male: 44 (20.8%)
Severino et al., 2011[56]	Brazil	Cohort	"To assess factors associated with reoperation and mortality in patients undergoing only conservative procedures in rheumatic mitral valve cause."	January 1994 - December 2005	Hospital (SC)		**X**		104	Surgical population<br>Mean age ± SD: 32.73 ± 14.74<br>Female: 82 (78.8%)<br>Male: 22 (21.2%)
Silva et al., 2010 [57]	Brazil	Cross-sectional	"To analyze the frequency of patients with RF admitted to Joana de Gusmão Children's Hospital, in Florianopolis, in southern Brazil, and their characteristics, between 1986 and 2006"	January - December of 1986 1991, 1996, 2001 and 2006	Hospital (SC)	**X**			1986: 4206<br>1991: 5206<br>1996: 5196<br>2001: 6777<br>2006: 8203<br>(*n of admissions)*<br><br>ARF cases: 99	ARF cases:<br>Mean age: 9.6<br>Female: 48 (48.5%)<br>Male: 51 (51.5%)
Soto Lopez et al., 2001 [58]	Mexico	Cross-sectional	"To assess the incidence of ARF "	1994 – 1999	Hospital (SC)<br>Population	**X**			Total ARF cases: 67<br>Total admissions (5–20-year-old): 3392	Mean age: 19.2<br>Female: 39 (58%)<br>28 (42%)
Souza et al., 1990 [59]	Brazil	Cross-sectional	"To estimate the occurrence of several serological groups of beta-hemolytic streptococci in clinical specimens of the oropharynx, in schoolchildren in the city of Rio de Janeiro and to describe the occurrence of RF and its different clinical forms and interrelationship between clinical and laboratory data."	N/A	School<br>Community	**X**			972	Age range: 3 – 15<br>Female: 457 (47%)<br>Male: 515 (53%)
Spitzer et al., 2015[60]	Peru	Cross-sectional	"To evaluate the implications of different classifications of RHD on estimated prevalence and to systematically assess the importance of incidental findings from echocardiographic screening among schoolchildren in Peru"	April 2014 - May 2014	School	**X**		**X**	1023	Median age (IQR): 11(8-13)<br>Female: 513 (50.1%)<br>Male: 510 (49.9%)
Stokes Baltazar, 2007 [61]	Guatemala	Cross-sectional	"To describe the incidence and characteristics of ARF in Izabal, Guatemala"	March 2000 - March 2005	Hospital (SC)	**X**			3422 (*n of admissions)*<br><br>ARF cases: 246	Female: 152 (61.8%)<br>Male: 94 (38.2%)
Travancas et al., 2009[62]	Brazil	Cohort	"To assess the outcomes in children and adolescents with RF of the implantation of mechanical	January 1996 - December 2005	Hospital (SC)		**X**		73	Surgical population<br>Mean age ± SD: 14.4 ± 2.96<br>Female: 34 (47.9%)

			as opposed to biological heart valves."							Male: 37 (52.1%)
Vasconcelos et al., 2021[63]	Brazil	Cohort	"To assess the incidence and predictors of stroke in a large cohort of patients with RHD on preventive strategies."	September 2010 - August 2019	Hospital (SC)		**X**		515	Mean age ± SD: 46 ± 12<br>Female: 438 (85%)<br>Male: 77 (15%)
WHO Cardiovascular Diseases Unit, 1992[64]	Bolivia<br>El Salvador<br>Jamaica	Cross-sectional	This paper presents the report from the Phase I of the WHO Programme for the prevention of ARF/RHD in 16 developing countries.	May 1986 - March 1990	School<br>Community	**X**		**X**	For prevalence data: 22,328	N/A
Zabal et al., 1992[65]	Mexico	Cohort	"To assess the long-term evolution of 155 patients under 16 years in whom a mechanical or biological prosthesis was implanted for mitral valve disease secondary to RF"	N/A	Hospital (SC)		**X**		155	Mean age:<br>- Group 1: 13.2<br>- Group 2: 13.6<br>- Group 3: 13.1<br>- Group 4: 12.3<br><br>Age range: 6 - 15

Abbreviations: AF: Atrial fibrillation; ARF: Acute rheumatic fever; Bur.: Burden of RHD; Cn: Net atrioventricular compliance; CVD: Cardiovascular Disease; DVR: Double Valve Replacement; Epi.: Epidemiology of Acute/RHD; HD: Heart diseases; ICD: International Classification of Diseases: IQR: Interquartile range; MC: Multicenter; MVR: Mitral Valve Replacement; MS: Mitral Stenosis; Prev./Scre.: Preventive/Screening strategies for RHD; PBMV: Percutaneous Baloon Mitral Valvuloplasty; PROVAR: Rheumatic Valve Disease Screening Program; PROVAR+: Programa de RastreamentO da VAlvopatia Reumática e outras Doenças Cardiovasculares; RCT: Randomized controlled trial; RF: Rheumatic fever; RHD: Rheumatic heart disease; RMVD: Rheumatic Mitral Valve Disease; SD: Standard deviation; SC: Single Cennter; UFMG: Federal University of Minas Gerais; WHO: World Health Organization

Table S6. Risk of bias assessment for cross-sectional studies (AXIS - Appraisal Tool for Cross-Sectional Studies [15])

	Q1	Q2	Q3	Q4	Q5	Q6	Q7	Q8	Q9	Q10	Q11	Q12	Q13	Q14	Q15	Q16	Q17	Q18	Q19	Q20	Tot.[1]
Alves Meira et al., 1995[18]	Yes	Yes	Yes	Yes	Yes	Yes	Yes	Yes	Yes	No	No	Yes	Yes	Yes	Yes	Yes	Yes	No	DK	Yes	15
Bach et al., 1996[19]	Yes	Yes	No	Yes	Yes	Yes	No	Yes	Yes	No	No	No	No	No	Yes	Yes	Yes	No	DK	DK	11
Beaton et al., 2016[20]	Yes	Yes	No	Yes	Yes	Yes	No	Yes	Yes	Yes	Yes	Yes	DK	No	Yes	Yes	Yes	Yes	No	Yes	16
Casalino et al., 2015[23]	Yes	Yes	No	Yes	Yes	Yes	Yes	Yes	Yes	Yes	Yes	Yes	No	Yes	Yes	Yes	Yes	Yes	No	Yes	19
de Araujo Fonseca et al., 2020[26]	Yes	Yes	No	Yes	Yes	Yes	No	Yes	Yes	Yes	Yes	No	No	No	Yes	Yes	Yes	Yes	No	Yes	16
Defilló Ricart et al., 1991 [27]	No	DK	No	Yes	Yes	Yes	No	Yes	Yes	No	No	No	DK	No	Yes	Yes	Yes	No	DK	DK	8
Fernandes et al., 2015 [29]	Yes	Yes	No	Yes	Yes	Yes	No	Yes	Yes	Yes	Yes	Yes	DK	No	Yes	Yes	Yes	No	No	Yes	15
Figuereido et al., 2019[30]	Yes	Yes	No	Yes	Yes	Yes	No	Yes	Yes	Yes	Yes	No	DK	No	Yes	Yes	Yes	Yes	No	Yes	15
Giachetto et al., 1994[31]	Yes	Yes	No	Yes	Yes	Yes	No	Yes	Yes	No	No	No	DK	No	No	Yes	Yes	No	DK	DK	9
Haddad and Silva, 2000[33]	Yes	Yes	No	Yes	Yes	Yes	No	Yes	Yes	No	Yes	Yes	No	No	Yes	Yes	Yes	Yes	DK	DK	14
Haddad and Bittar, 2005 [32]	Yes	Yes	No	Yes	Yes	Yes	DK	Yes	Yes	Yes	Yes	No	DK	No	Yes	Yes	Yes	Yes	No	DK	14
Isaacura and Granero, 1998[34]	Yes	Yes	No	Yes	Yes	Yes	No	Yes	Yes	No	Yes	No	No	No	Yes	Yes	Yes	No	DK	DK	12
Lolio et al., 1991[37]	Yes	Yes	No	Yes	Yes	Yes	No	Yes	Yes	No	Yes	Yes	No	No	Yes	Yes	Yes	No	No	DK	14
Luque et al., 2006[38]	Yes	Yes	No	Yes	Yes	Yes	No	Yes	Yes	No	No	No	No	No	Yes	Yes	Yes	No	No	DK	12
Millard-Bullock, 2012[42]	No	DK	No	Yes	Yes	Yes	No	Yes	Yes	No	No	No	DK	No	Yes	Yes	Yes	No	DK	DK	8
Miranda et al., 2014[43]	Yes	Yes	Yes	Yes	Yes	Yes	DK	Yes	Yes	Yes	Yes	Yes	DK	DK	Yes	Yes	Yes	Yes	No	Yes	17
Nascimento et al., 2021[45]	Yes	Yes	Yes	Yes	Yes	Yes	Yes	Yes	Yes	Yes	Yes	Yes	Yes	Yes	Yes	Yes	Yes	Yes	No	Yes	19
Nascimento, Sable et al., 2018[46]	Yes	Yes	No	Yes	Yes	Yes	No	Yes	Yes	Yes	Yes	Yes	Yes	No	Yes	Yes	Yes	Yes	No	Yes	16
Noah et al., 1994[47]	Yes	Yes	No	Yes	Yes	Yes	No	Yes	Yes	No	No	No	DK	No	Yes	Yes	Yes	Yes	DK	DK	11
Nordet et al., 2008[48]	Yes	Yes	No	Yes	Yes	Yes	No	Yes	Yes	No	Yes	No	No	No	Yes	Yes	Yes	No	DK	DK	12
Paar et al., 2010[51]	Yes	Yes	Yes	Yes	Yes	Yes	No	Yes	Yes	Yes	Yes	Yes	No	No	Yes	Yes	Yes	No	No	Yes	17
Pato et al., 2015[52]	Yes	Yes	No	Yes	Yes	Yes	No	Yes	Yes	Yes	Yes	Yes	No	No	Yes	Yes	Yes	Yes	No	Yes	17
Ribeiro et al., 2012[53]	Yes	Yes	No	Yes	Yes	Yes	No	Yes	Yes	Yes	Yes	Yes	No	No	Yes	Yes	Yes	No	No	Yes	16
Salinas Mondragón et al., 1995[54]	Yes	Yes	No	Yes	Yes	Yes	No	Yes	Yes	No	Yes	Yes	DK	No	Yes	Yes	Yes	No	DK	DK	12
Silva et al., 2010[57]	Yes	Yes	No	Yes	Yes	Yes	No	Yes	Yes	No	Yes	Yes	No	No	Yes	Yes	Yes	Yes	No	Yes	16
Soto López et al., 2001[58]	Yes	Yes	No	Yes	Yes	Yes	No	Yes	Yes	No	No	Yes	DK	No	Yes	Yes	Yes	No	DK	DK	11
Souza et al., 1990[59]	Yes	Yes	No	Yes	Yes	Yes	No	Yes	Yes	No	No	Yes	DK	No	Yes	Yes	Yes	No	No	DK	12
Spitzer et al., 2015[60]	Yes	Yes	Yes	Yes	Yes	Yes	Yes	Yes	Yes	Yes	Yes	Yes	No	Yes	Yes	Yes	Yes	Yes	No	Yes	20

Stokes Baltazar, 2007[61]	No	Yes	No	Yes	Yes	Yes	No	Yes	Yes	No	No	Yes	DK	No	Yes	Yes	Yes	Yes	DK	DK	11
WHO Cardiovascular Diseases Unit, 1992[64]	Yes	Yes	No	Yes	Yes	Yes	No	Yes	Yes	No	No	No	DK	No	Yes	Yes	Yes	No	No	DK	11

[1]Total score ranges from 0 – 20. A point was awarded for each positive aspect ("Yes" for Q1-12,14-18, and 20; "No" for Q13 and 19)

Abbreviations: DK: Don't know, Q: Question, Tot.:Total

Table S7. Risk of bias assessment for cohort studies (The Newcastle Ottawa Scale [14])

	**Selection[1]**	**Comparability[1]**	**Outcome[1]**	**Total[1]**
Berrios et al., 1993 [21]	✵✵✵		✵✵	**5**
Canale et al., 2011[22]	✵✵✵		✵✵	**5**
Chavez et al., 2017[24]	✵✵✵		✵✵✵	**6**
Cruz et al., 2019[25]	✵✵✵		✵✵	**5**
Durães et al., 2013[28]	✵✵✵✵	✵✵	✵✵✵	**9**
Jatene et al., 2000[35]	✵✵✵✵	✵✵	✵✵✵	**9**
Meira et al., 2005 [39]	✵✵✵		✵✵✵	**6**
Mejia et al., 2018[40]	✵✵✵✵		✵✵✵	**7**
Meneguz-Moreno et al., 2018[41]	✵✵✵	✵	✵✵	**6**
Mota et al., 2015[44]	✵✵✵✵	✵✵	✵✵✵	**9**
Nunes et al., 2013[49]	✵✵✵		✵✵✵	**6**
Seguel et al., 2008 [55]	✵✵✵✵	✵✵	✵✵	**8**
Severino et al., 2011 [56]	✵✵✵		✵✵✵	**6**
Travancas et al., 2009 [62]	✵✵✵✵	✵	✵✵✵	**8**
Vasconcelos et al., 2021 [63]	✵✵✵✵		✵✵✵	**7**
Zabal et al., 1992[65]	✵✵✵✵	✵	✵✵✵	**8**

[1]Selection: 0-4 stars; Comparability: 0-2 stars; Outcome: 0-3 stars; Total 0-9 stars

Table S8. Risk of bias assessment for randomized controlled trials (The Cochrane RoB 2.0 tool and The Cochrane RoB 2.0 tool for Cluster Randomized Trials[16,17]**)**

	Randomization Process	Timing of identification or recruitment of participants in a cluster-randomized trial*	Deviations from Intended Interventions	Missing Outcome Data	Measurement of the Outcome	Selection of the Reported Result	Overall Bias
Lavitola et al., 2010[1] [36]	**Some concerns**	**N/A**	**Some concerns**	**Low**	**Low**	**Some concerns**	**Some concerns**
Oliveira et al, 2020[2] [50]	**Some concerns**	**Low**	**Some concerns**	**Low**	**Low**	**Some concerns**	**Some concerns**

*****Only included in the Cochrane RoB 2.0 tool for Cluster Randomized Trials; just applicable to Oliveira et al. 2020 [50]
[1]Assessed using The Cochrane RoB 2.0 tool (introduced in 2016 and last edited on August 22, 2019)
[2]Assessed using The Cochrane RoB 2.0 tool for Cluster Randomized Trials (introduced in 2020 and last edited on March 18, 2021)

Abbreviations: N/A: Not Applicable.

Supplementary Table S9. Burden of Rheumatic Heart Disease per included studies

Reference	Country	Period	n	Age	Burden of RHD[6]							
					Mortality	Need for intervention[1]	HF	AFib	IE	Stroke	EE	PH
Figueiredo et al., 2019 [30]	BR	1998-2016	N/A	N/A	1998: 5.77<br>2016: 8.22	NR	NR	NR	NR	NR	NR	NR
Haddad and Silva, 2000[2][33]	BR	1991-1995	N/A	Range: 15 – 49	1.58/100,000 women	NR	NR	NR	NR	NR	NR	NR
Lavitola et al., 2010 [36]	BR	N/A	229	N/A	NR	NR	NR	NR	NR	29 (12.7%)	10 (4.4%)	NR
Lolio et al., 1991[2] [37]	BR	1986	953	Range: 10-49	2.6 per 100,000 women (15-44 years)	NR	NR	NR	NR	NR	NR	NR
Nunes et al., 2013 [49]	BR	2007-2011	128	Mean ± SD: 42.6 ± 11.2	Follow up (22-month): 1 (0.8%)	Baseline: 35 (27%)<br>Follow-up: 44 (34.4%)	NR	18 (14%)	NR	NR	NR	NR
Vasconcelos et al., 2021 [63]	BR	2010-2019	515	Mean ± SD: 46 ± 12	32 (6.2%)	Prior PMV: 130 (25%)<br><br>Follow-up: 105 (21.5%)	NR	155 (30%)	NR	Baseline: 92 (18%)<br><br>Follow-up:<br>27 (5.2%)<br><br>*Incidence: 1.47 strokes per 100 patient-years*	NR	NR
Nordet et al., 2008 [48]	CU	1986-1996	134 *(1986-90)*<br><br>193 *(1991-96)*	Range: 5-25	NR	1986-1990: 6 (4.5%)<br><br>1991-1996: 1 (0.5%)<br><br>1986: 1/52 (1.9%)<br><br>1996: 0	1986-1990: 15 (11.2%)<br><br>1991-1996: 3 (1.5%)<br><br>1986: 5/52 (9.6%)<br><br>1996: 0	NR	1986-1996: 0	NR	NR	NR
Salinas Mondragón et al., 1995 [54]	PE	1989-1993	66	N/A	4 (6%)	8 (12.1%)	NR	NR	15 (23%)	1 (1.5%)	NR	11 (16.7%)

Isaacura and Granero, 1998 [34]	VE	1955-1994	N/A	N/A	1955: 7.06<br>1966: 3.04<br>1975: 0.78<br>1985: 1.66<br>1994: 1.05<br><br>*Rates per 100,000*	NR	NR	NR	NR	NR	NR	NR
***Surgical/Interventional only*[3]**												
Canale et al., 2011 [22]	BR	2008-2009	53	Mean ± SD: 49.3± 10.7	Intraoperative: 7 (13%)	NR	NR	NR	Baseline: 1 (1.9%)	Baseline: 4 (7.5%)	NR	NR
Casalino et al., 2015 [23]	BR	2009	247	Mean ± SD: 51 ± 12	NR	NR	55 (22.3%)[3]	Baseline: 131 (53.1%)	NR	NR	NR	142 (57.5%)[5]
Chavez et al., 2017[24]	BR	2013-2014	103	Mean ± SD: 50.76 ± 10.7	< 30 days: 8 (7.8%)<br><br>Late (1-year): 0	NR	NR	NR	NR	Baseline: 11 (10.5%)<br>Postop.: 1 (1%)	NR	NR
Cruz et al., 2019[25]	BR	2011-2017	54	Mean ± SD:10.5 ± 3.2	2-month: 0	Reintervention: 3 (5.6%)	Postoperative: 4 (7.4%)	0	NR	NR	NR	Preop.: 38 (77.6%)[5]<br>Postop.: 7 (18.4%)[5]
Durães et al., 2013 [28]	BR	2010-2012	184	Mean ± SD:<br>45 ± 16<br>42 ± 15	3-month: 0	NR	NR	NR	NR	5 (2.7%)	NR	NR
Fernandes et al., 2015 [29]	BR	2007-2011	104	Mean ± SD: 38.04 ± 14.4	In-hospital: 20 (19.2%)	Prior surgery**:** 38%<br><br>Reintervention**:** 24 (23.07%)	NR	28/100 (28%)	NR	NR	NR	NR
Jatene et al., 2000[35]	BR	1991-1994	55	Mean: 51.5	30 days: 3 (5.4%)<br><br>Follow-up (38.5 & 41.1 months): 4 (7.3%)	NR	NR	NR	NR	NR	Baseline: 9 (16.4%)<br><br>Postop.: 7 (12.7%)	NR
Mejia et al., 2018[40]	BR	2010-2015	2919	Mean ± SD:<br>51.2 ± 14.9<br>54.4 ± 17.4	In-hospital/<30 days:<br>99 (3.51%)	Reoperation:<br>**-First**: 813 (27.9%)<br>**-Second**: 432 (14.8%)	NR	NR	NR	NR	NR	NR

Meneguz-Moreno et al., 2018[41]	BR	1987-2010	1582	Mean ± SD: 36.44 ± 12.77	0.6%	After initial PBMV:<br>**-Mitral surgery:** 104 (8.3%)<br>**-PBMV:** 125 (10%)	NR	198 (12.5%)	NR	NR	NR	NR
Pato et al., 2015 [52]	BR	2010-2011	32	Mean ± SD: 45.9 ± 14.0	In-hospital: 3 (10%)	Baseline: 20 (63%)<br><br>Reoperation: 3 (10%)	NR	NR	5 (16%)	NR	NR	NR
Ribeiro et al., 2012 [53]	BR	2002-2005	296	Median (IQR): 37 (25 - 48)	In-hospital: 26 (9%)<br><br>*Mean annual in-hospital due to RHD surgery:* 0.25 per 100,000	Baseline: 89 (30%)<br><br>*Mean annual incidence of open-heart surgery for RHD:* 2.86 per 100,000	NR	NR	NR	NR	NR	NR
Severino et al., 2011 [56]	BR	1994-2005	104	Mean ± SD: 32.73 ± 14.74	Operative & In-hospital: 0<br><br>Long-term (63±39-month): 3 (2.9%)	Reoperation: 12 (11.5%)	NR	NR	NR	NR	NR	NR
Travancas et al., 2009[62]	BR	1996-2005	73	Mean ± SD: 14.4 ± 2.96	Operative: 2 (2.7%)<br><br>Overall: 6 (8.2%)	Reoperation: 9/71 (12.7%)	NR	NR	2/71 (2.8%)	3/71 (4.2%)	NR	NR
Seguel et al., 2008[55]	CL	1990-2004	212	Mean: 45.53 43.66	Operative: 20 (9.4%)<br><br>Follow-up (6.67-7.89 years): 34/192 (17.7%)	Reoperation: 10 (4.7%)	11 (5.1%)	Preop.: 139 (65.6%)<br><br>Postop.: 100/158 (63.3%)	3 (1.4%)	6 (2.8%)	NR	NR
Zabal et al., 1992[65]	MX	N/A	155	Range: 6-15	31 (20%)	NR	NR	NR	11 (7.1%)	NR	11 (7.1%)	NR

[1] "Need for intervention" includes any surgical (initial or reoperation) or percutaneous intervention (initial or reintervention).
[2] Studies that assessed data on female population of childbearing age only.
[3] Studies that assessed solely surgical or percutaneously intervened RHD patients.
[4] Reported as impaired left ventricular ejection fraction on echocardiography only.
[5] Pulmonary hypertension diagnosis was based on echocardiographic findings.
[6] Additional variables on burden of RHD:

1. **Need for hospitalization:** Nordet et al. [48]: 1986-1990: 55/134 (41.4%), 1991-1996: 16/193 (8.3%)
2. **Need for anticoagulation:** Vasconcelos et al. [63]: 183 (36%); Pato et al. [52]: 15 (60%)
3. **Anticoagulated-related complications:** Lavitola et al. [36]: Gynecological or gastrointestinal bleeding: 44/119 (36.9%); Travancas et al. [62]: Bleeding: 7/71 (9.9%); Zabal et al. [65]: Intracranial hemorrhage leading to death: 1/36 (2.8%)

Abbreviations: AFib: Atrial fibrillation; BR: Brazil; CL: Chile; CU: Cuba; EE: Embolic events; IE: Infective endocarditis; IQR: Interquartile Range; N/A: Not available; NR: Not reported; MX: Mexico; PBMV: Percutaneous Balloon Mitral Valvuloplasty; PE: Peru; PH: Pulmonary hypertension; PMV: Percutaneous Mitral Valvuloplasty; Preop.: Preoperative; Postop.: Postoperative; RHD: Rheumatic Heart Disease; SD: Standard Deviation; VE: Venezuela

Section and Topic	Item #	Checklist item	Location where item is reported
**TITLE**			
Title	1	Identify the report as a systematic review.	Pg. 1
**ABSTRACT**			
Abstract	2	See the PRISMA 2020 for Abstracts checklist.	Pg. 2
**INTRODUCTION**			
Rationale	3	Describe the rationale for the review in the context of existing knowledge.	Pg. 3
Objectives	4	Provide an explicit statement of the objective(s) or question(s) the review addresses.	Pg. 3
**METHODS**			
Eligibility criteria	5	Specify the inclusion and exclusion criteria for the review and how studies were grouped for the syntheses.	Pg. 4
Information sources	6	Specify all databases, registers, websites, organisations, reference lists and other sources searched or consulted to identify studies. Specify the date when each source was last searched or consulted.	Pg. 4
Search strategy	7	Present the full search strategies for all databases, registers and websites, including any filters and limits used.	Sup Table S1 – S4
Selection process	8	Specify the methods used to decide whether a study met the inclusion criteria of the review, including how many reviewers screened each record and each report retrieved, whether they worked independently, and if applicable, details of automation tools used in the process.	Pg. 4
Data collection process	9	Specify the methods used to collect data from reports, including how many reviewers collected data from each report, whether they worked independently, any processes for obtaining or confirming data from study investigators, and if applicable, details of automation tools used in the process.	Pg. 4-5
Data items	10a	List and define all outcomes for which data were sought. Specify whether all results that were compatible with each outcome domain in each study were sought (e.g. for all measures, time points, analyses), and if not, the methods used to decide which results to collect.	Pg. 4-5
	10b	List and define all other variables for which data were sought (e.g. participant and intervention characteristics, funding sources). Describe any assumptions made about any missing or unclear information.	Pg. 4-5
Study risk of bias assessment	11	Specify the methods used to assess risk of bias in the included studies, including details of the tool(s) used, how many reviewers assessed each study and whether they worked independently, and if applicable, details of automation tools used in the process.	Pg. 5
Effect measures	12	Specify for each outcome the effect measure(s) (e.g. risk ratio, mean difference) used in the synthesis or presentation of results.	N/A
Synthesis methods	13a	Describe the processes used to decide which studies were eligible for each synthesis (e.g. tabulating the study intervention characteristics and comparing against the planned groups for each synthesis (item #5)).	Pg. 5
	13b	Describe any methods required to prepare the data for presentation or synthesis, such as handling of missing summary statistics, or data conversions.	Pg. 5
	13c	Describe any methods used to tabulate or visually display results of individual studies and syntheses.	Pg. 5
	13d	Describe any methods used to synthesize results and provide a rationale for the choice(s). If meta-analysis was performed, describe the	Pg. 5

		model(s), method(s) to identify the presence and extent of statistical heterogeneity, and software package(s) used.	
	13e	Describe any methods used to explore possible causes of heterogeneity among study results (e.g. subgroup analysis, meta-regression).	N/A
	13f	Describe any sensitivity analyses conducted to assess robustness of the synthesized results.	N/A
Reporting bias assessment	14	Describe any methods used to assess risk of bias due to missing results in a synthesis (arising from reporting biases).	N/A
Certainty assessment	15	Describe any methods used to assess certainty (or confidence) in the body of evidence for an outcome.	N/A
**RESULTS**			
Study selection	16a	Describe the results of the search and selection process, from the number of records identified in the search to the number of studies included in the review, ideally using a flow diagram.	Pg. 5, Fig 1
	16b	Cite studies that might appear to meet the inclusion criteria, but which were excluded, and explain why they were excluded.	Pg. 5, Fig 1
Study characteristics	17	Cite each included study and present its characteristics.	Pg. 5, Pg. 6, Sup Table S5
Risk of bias in studies	18	Present assessments of risk of bias for each included study.	Pg. 6, Sup Table S6-S8
Results of individual studies	19	For all outcomes, present, for each study: (a) summary statistics for each group (where appropriate) and (b) an effect estimate and its precision (e.g. confidence/credible interval), ideally using structured tables or plots.	Tables 1 – 4, Sup Table S9
Results of syntheses	20a	For each synthesis, briefly summarise the characteristics and risk of bias among contributing studies.	Pg. 6 – 9, Tables 1 – 4
	20b	Present results of all statistical syntheses conducted. If meta-analysis was done, present for each the summary estimate and its precision (e.g. confidence/credible interval) and measures of statistical heterogeneity. If comparing groups, describe the direction of the effect.	Pg. 6 – 9, Tables 1 – 4
	20c	Present results of all investigations of possible causes of heterogeneity among study results.	N/A
	20d	Present results of all sensitivity analyses conducted to assess the robustness of the synthesized results.	N/A
Reporting biases	21	Present assessments of risk of bias due to missing results (arising from reporting biases) for each synthesis assessed.	N/A
Certainty of evidence	22	Present assessments of certainty (or confidence) in the body of evidence for each outcome assessed.	N/A
**DISCUSSION**			
Discussion	23a	Provide a general interpretation of the results in the context of other evidence.	Pg. 10 - 13
	23b	Discuss any limitations of the evidence included in the review.	Pg. 12 – 13
	23c	Discuss any limitations of the review processes used.	Pg. 12 – 13
	23d	Discuss implications of the results for practice, policy, and future research.	Pg. 13

OTHER INFORMATION			
Registration and protocol	24a	Provide registration information for the review, including register name and registration number, or state that the review was not registered.	Pg. 4
	24b	Indicate where the review protocol can be accessed, or state that a protocol was not prepared.	Pg. 4 and Reference #12
	24c	Describe and explain any amendments to information provided at registration or in the protocol.	N/A
Support	25	Describe sources of financial or non-financial support for the review, and the role of the funders or sponsors in the review.	Pg. 13
Competing interests	26	Declare any competing interests of review authors.	Pg. 13
Availability of data, code and other materials	27	Report which of the following are publicly available and where they can be found: template data collection forms; data extracted from included studies; data used for all analyses; analytic code; any other materials used in the review.	Pg. 13

From: Page MJ, McKenzie JE, Bossuyt PM, Boutron I, Hoffmann TC, Mulrow CD, et al. The PRISMA 2020 statement: an updated guideline for reporting systematic reviews. BMJ 2021;372:n71. doi: 10.1136/bmj.n71

For more information, visit: http://www.prisma-statement.org/
